# Neuronal Sik1 in the Hypothalamic Paraventricular Nucleus Decreases Blood pressure Elevation Following a High-Salt Diet

**DOI:** 10.1007/s12035-026-05666-6

**Published:** 2026-01-16

**Authors:** Wei Zhang, Ping Wang, Shuya Qi, Na Huang, Qingyun Huang, Zhongxin Guo, Weifeng Wu, Guohe Tan

**Affiliations:** 1 Institute of Neuroscience and Guangxi Key Laboratory of Brain Science, Department of Human Anatomy, School of Basic Medical Sciences, Guangxi Medical University, 22 Shuang-Yong Road, Nanning, Guangxi 530021 People’s Republic of China; 2 Department of Cardiology, The First Affiliated Hospital of Guangxi Medical University, Nanning, Guangxi China; 3 Key Laboratory of Longevity and Aging-Related Diseases of Chinese Ministry of Education, Guangxi Health Commission Key Laboratory of Basic Research On Brain Function and Disease, Nanning, Guangxi China; 4 Collaborative Innovation Centre of Regenerative Medicine and Medical BioResource Development and Application Co-Constructed By the Province and Ministry, Key Laboratory of Regenerative Medicine, Guangxi Medical University, Nanning, Guangxi China; 5 Department of Neurology, Guangxi Hospital Division of the First Affiliated Hospital of Sun Yat-sen University, Nanning, Guangxi, China

**Keywords:** Sik1, Paraventricular nucleus, Blood pressure, High-salt diet

## Abstract

Blood pressure (BP) regulation involves complex interactions between peripheral organs and the brain. As a key area gating BP regulation, how the hypothalamic paraventricular nucleus (PVN) modulates salt‑sensitive hypertension remains unclear. Here, we found that Sik1, a member of the AMP-activated protein kinase family, was upregulated in PVN neurons of mice following a high-salt diet (HSD). When Sik1 was ablated, *Sik1* knockout mice exhibited an increase in BP upon HSD feeding. Furthermore, specific deletion of the *Sik1* gene in the nervous system by *Nestin-Cre* (*Nestin-Cre;Sik1*^–/–^) resulted in elevated BP after high salt intake. Notably, AAV-Cre-mediated selective ablation of Sik1 in the PVN neurons was sufficient to cause BP elevation following an HSD. In combination with western blot and immunofluorescence detection, single-nucleus RNA sequencing combined with KEGG pathway analysis showed that Sik1 is predominantly expressed in arginine vasopressin (AVP)-positive neurons of the PVN, and in the absence of Sik1, the cellular NF-κB pathway in these neurons is downregulated by HSD. In addition, Sik1 deficiency led to microglial activation within the PVN under HSD conditions. These results suggest that Sik1 in AVP-positive neurons of PVN attenuates HSD-induced blood pressure elevation, possibly via modulation by the NF-κB signaling pathway. Our findings uncover a previously unrecognized role of neuronal Sik1 in salt‑sensitive hypertension pathophysiology, advancing our understanding of neurogenic blood pressure regulation.

**Supplementary Information:**

The online version contains supplementary material available at 10.1007/s12035-026-05666-6.

## Introduction

Blood pressure, determined by the dynamic balance between cardiac output and vascular resistance, is regulated by the cardiovascular system, kidneys, and neurohormonal mechanisms, including the sympathetic nervous system (SNS) and renin–angiotensin–aldosterone system [1, 2]. The SNS, influenced by brain regions like the hypothalamus and rostral ventrolateral medulla, modulates heart rate, vasoconstriction, and sodium re-absorption [3]. Recently, the central nervous system, particularly the hypothalamus, has emerged as a key regulator of BP. The PVN of the hypothalamus integrates neuroendocrine and autonomic signals to control BP and cardiovascular function [4, 5]. Studies have reported that neuroinflammation in the PVN can drive sympathetic overactivation, leading to hypertension [6–8]. Recent evidence further suggests that PVN dysfunction is also associated with cardiovascular and metabolic disorders [9–11]. Despite its established role, the precise molecular mechanisms within the PVN underlying ​salt-induced BP elevation​ remain unclear.

Excessive salt intake is considered a risk factor for elevated BP [12, 13]. A series of experimental and clinical studies have shown that an HSD increases the concentration of sodium ions ([Na^+^]) in both plasma and cerebrospinal fluid (CSF) [14–16]. Elevated sodium ion concentration in the cerebrospinal fluid has been proven to enhance SNS excitability and stress responses in salt-sensitive rats [17]. Additionally, long-term high salt intake has been found to alter neuronal activity in the PVN [18, 19]. Salt-inducible kinase 1 (Sik1), which encoding gene discovered in the adrenal glands of rats consuming an HSD, is a member of the AMP-activated protein kinase (AMPK) family [20, 21]. Emerging evidences have shown that Sik1 in neurons plays a crucial regulatory role in [Na^+^] transport mechanisms within the central nervous system [17]. In human genetic studies, single nucleotide polymorphisms in the *SIK1* gene have been associated with hypertension [22]. Recent studies have found that Sik1 is also expressed in the brain and can respond to neuronal activity [23, 24]. However, the potential role of Sik1 within the PVN in salt-induced BP elevation is still obscure.

This research aimed to provide new clues about the role of Sik1 in BP regulation via the PVN. This study used *Sik1* mutant mice in HSD models to explore Sik1’s involvement in the PVN’s regulation of BP. Our findings show that Sik1 expression in the PVN is upregulated following the consumption of an HSD. Furthermore, the ablation of Sik1 in mice fed with an HSD leads to an increase in BP. To avoid cardiovascular confounding factors, we specifically ablated Sik1 in the nervous system that resulted in elevated BP upon high salt intake. Importantly, the PVN is a key area mediating Sik1’s function in regulating BP under an HSD, as revealed by the observation that selectively knockout of *Sik1* in the mouse PVN was sufficient to cause salt-induced BP elevation. Notably, we found that the NF-κB p65 signaling pathway is involved in the regulation of BP by Sik1 in the PVN upon high salt intake. These findings underscore the mechanisms by which Sik1 in the PVN regulates BP in response to an HSD, offering potential insights into the neurogenic mechanisms underlying BP elevation following high salt intake.

## Methods

### Experimental Animals

*Sik1* knockout mice with a C57BL/6 J background were generated using CRISPR/Cas-mediated genome editing in accordance with previously described protocols [25]. Briefly, the Cas9 mRNA was transcribed in vitro by mMACHINE ® T7 Ultra Kit, the guide RNA (Site 1: Sik1 intron3, 5′-GCAATCCCCTGTAGCTTACC-3′, 5′- GCCTAATGTCTTGTCAGTAG-3′ primers, Site 2: Sik1 intron7, 5′-TTAGCTTTGCAATCGACAAT-3′, 5′-CTCCTGTTGTGGTACACGTG-3′ primers) was transcribed in vitro by MEGAshortscript™ Kit and then purified using a MEGAclearTM Kit (Invitrogen). Subsequently, mixtures (100 μl) were microinjected into the male pronucleus of fertilized eggs; the eggs were then transferred into the oviducts of pseudopregnant C57BL/6 J female mice. After site-specific genome modification analysis using PCR and sequencing, 38 viable pups were born, of which 13 were identified to carry the correct mutant allele. Two F0 mice were selected for crossing with wild-type (WT) mice with the same C57BL/6 J background to produce the F1 generation. I﻿n brief, the targeting construct used for homologous recombination at the *Sik1* locus in murine embryonic stem (ES) cells was cloned in two arms by PCR amplification of C57BL/6 J genomic DNA. The 5′ arm was PCR-amplified with 5′-GGTCCCTGAAGGTAGTCAGCGTTTCTA-3′ and 5′-CTCACAGACCTGAAGTGGCATAGCCTT-3′ primers, whereas the 3′ arm was PCR-amplified with 5′-GGCTGTGTGGGAACTGAACACAGAG −3′ and 5′-CTCCTTCTGGCTCTTTCTGGCAGTTACT −3′ primers. Exon 4–7 encoding the core region of murine *Sik1* was flanked with a pair of *loxP* sequences (*Sik1*^loxP/loxP^), and a neomycin resistance cassette (Neo) flanked with a pair of FRT sites was used for positive selection. The Neo cassette was ultimately removed by crossing with transgenic mice (SBOSTD Co., Ltd., China) carrying Flp recombinase prior to crossing with Cre transgenic mice. The *loxP* sites do not interfere with the normal expression of the target gene but constitute a binding domain for the DNA recombinase Cre. The Nestin-Cre transgenic mouse line were purchased from SBOSTD Co., Ltd., China. As previous reported [26, 27], the mice expressed Cre recombinase under the control of the neural-specific nestin promoter, which directs gene deletion specifically within the central nervous system, while excluding expression in non-neural tissues. Using *Cre-loxP* technology, we crossed *Nestin-Cre* mice with *Sik1*^flox/flox^ mice to generate *Sik1* conditional knockout (cKO) mice. Both *Sik1* knockout mice and *Sik1*-floxed mice were generated by Shanghai Biomodel Organism Science & Technology Development (SBOSTD) Co., Ltd.

We maintained the mice in a specific pathogen-free (SPF)-grade animal room at room temperature with a 12 h/12 h light/dark cycle with free access to food and water. Age-and gender-balanced littermates were randomly assigned to the experimental groups. At 8 weeks of age, all mice underwent a 5-day adaptation period on a normal-salt diet (NSD) (containing 0.3% NaCl), followed by a 7-day challenge with either an HSD (containing 8% NaCl) or an NSD. Tests were conducted during the day cycle between 9:00 a.m. and 2:00 p.m. The experimenters were blinded to both the genotypes and treatments of the mice. Both male and female animals were examined in this study, and similar findings were reported for both sexes.

### Blood Pressure Measurements

Systolic blood pressure (SBP) was measured once daily during both the 5-day adaptation period and the 7-day molding period, using a standard tail cuff system (BP-2010A system, Softron) [28]. SBP was measured three times for each mouse, and the average of the three readings was calculated. Measurements were taken between 9:00 a.m. and 2:00 p.m. each day.

### RNA Extraction, cDNA Synthesis, Reverse-Transcription PCR, and Real-Time PCR

The hypothalamus was collected to detect the expression level of the indicated genes after 7 days of HSD feeding. Total RNA was extracted from the mouse hypothalamus using TRIzol reagent (Invitrogen, USA). A Revert Aid First Strand cDNA Synthesis Kit (Thermo Scientific, USA) was used to reverse transcribe total RNA into cDNA, and SYBR Green Master Mix (Vazyme, China) was used for real-time PCR. We used *Gapdh* as a control RNA. We used the same primers as those used for the RT-PCR. The primers used in the experiments: *Gapdh*, Forward primer: GGTTGTCTCCTGCGACTTCA, Reverse primer: CCACCACCCTGTTGCTGTAG; *Sik1*, Forward primer: ACGGGCACTTGAGTGAAAAC, Reverse primer: TTCCCAAATCCAAAATCTGC.

### Western Blot

Tissues were homogenized using RIPA buffer containing protease inhibitors, followed by centrifugation at 12,000 rpm for 20 min at 4 °C to remove debris. Protein concentrations were measured using the bicinchoninic acid protein assay kit and were denatured by heating at 100 °C for 10 min. The resulting protein samples were transferred to PVDF membranes from the SDS-PAGE gel and then were incubated in TBST containing 5% skim milk or 5% bovine serum albumin (BSA) for 1 h at room temperature before overnight primary antibody incubation at 4 °C. Then, the membranes were incubated with the secondary antibodies for 2 h. After incubation with enhanced chemiluminescence, immunoreactive bands were visualized using Bio-RAD automatic imaging system scanners. The intensity of the immunoreactive bands was quantitated using ImageJ, with GAPDH (KC-5G4, 1:10,000, Aksomics) as the loading control. For primary antibodies, we used rabbit anti-Sik1 (1:500, Willget biotech), rabbit anti-IL-1β antibody (TA5103, 1:500, Abmart), rabbit anti-IĸB-α antibody (T55026, 1:500, Abmart), rabbit anti-NF-κB p65 antibody (T55034, 1:500, Abmart), rabbit anti-p-NF-κB p65 antibody (TP56372, 1:500, Abmart), goat anti-IBA1 antibody (ab5670, 1:500, Abcam); For the secondary antibodies, we used goat anti-rabbit (KC-RB-035, 1:10,000, Aksomics) or donkey anti-goat (KC-GT-035, 1:10,000, Aksomics) horseradish peroxidase (HRP)-conjugated secondary antibodies.

### Immunofluorescence, Immunohistochemistry, and Nissl Staining

Mice were anesthetized by inhalation of isoflurane and perfused with saline, followed by 4% paraformaldehyde (PFA). Their brains were postfixed in 4% PFA for 10–12 h at 4 °C and transferred to 15% and 30% sucrose in phosphate buffer for 10–12 h at 4 °C. Samples were embedded in optimal cutting temperature (O.C.T.) medium (SAKURA). Frozen blocks were sliced with a cryostat (Leica, CM1950) to prepare 30 μm-thick slices.

For immunofluorescence, slices were washed with 1 × phosphate buffered saline (PBS) and blocked with 1 × PBS containing 1% Triton-100 and 10% donkey serum for 1 h at room temperature, before overnight incubation of rabbit anti-Sik1 antibody (1:200, Willget biotech), mouse anti-NeuN antibody (1:500, MAB377, Merck Millipore), chicken anti-GFAP (1:500, ab7260, Abcam), goat anti-Iba1 antibody (1:500, ab5670, Abcam), mouse anti-Cre (1:500, MAB3120, Millipore), rabbit anti-Vasopressin antibody (1:500, 20,069, immunostar), rabbit anti-Oxytocin antibody (1:500, 20,068, immunostar), rabbit anti- CRF antibody (1:500, ab272391, Abcam), rabbit anti-IL-1β antibody (1:100, Abmart, TA5103), rabbit anti-p-NF-κB p65 antibody (Abmart, TP56372). After washing in 1 × PBS three times, the slices were incubated in Alexa Fluor 488-conjugated donkey anti-rabbit IgG H&L (A21206, 1:1000, Thermo Fisher Scientific), Alexa Fluor 568-conjugated donkey anti-rabbit IgG H&L (A10042, 1:1000, Thermo Fisher Scientific), Alexa Fluor 546-conjugated goat anti-chicken IgG H&L (A11040, 1:1000, Thermo Fisher Scientific), Alexa Fluor 647-conjugated goat anti-mouse IgG H&L (A21236, 1:1000, Thermo Fisher Scientific), and Alexa Fluor 647-conjugated donkey anti-goat IgG H&L (A21447, 1:1000, Thermo Fisher Scientific) for 2 h at room temperature. After washing in 1 × PBS three times, the slices were counterstained with Hoechst (#33,342, 1:8000, Beyotime). The slices were only incubated in the secondary antibodies as a negative control. We visualized the samples using confocal laser scanning microscopy (Leica).

For immunohistochemistry (IHC) staining, an UltraSensitiveTM SP rabbit IHC Kit (KIT-9706, MXB biotechnologies) and a DAB staining kit (KIT-5220, MXB biotechnologies) were used according to the manufacturer's protocol. After antigen retrieval, brain sections were incubated with endogenous peroxidase blocking solution for 10 min at room temperature. Brain sections were blocked with goat serum for 10 min at room temperature and then incubated at 4 °C with the anti-Sik1 primary antibodies (1:200, Willget biotech) overnight. Brain sections were further incubated with biotinylated secondary antibodies for 10 min at room temperature and then with streptavidin-peroxidase. To visualize the signals, the sections were incubated with DAB solution for 20 s, followed by rinsing in 1 × PBS to stop the reaction.

The brain sections were stained with a Nissl stain (Toluidine, Sinopharm Chemical Reagent Co., LTD) for 10 s at room temperature. After washing in double-distilled water for 10 s, gradient ethanol dehydration was performed on the brain sections. Images were collected using Leica DMi4 microscopy (Leica).

### Stereotaxic Injection of AAV

To achieve localized knockout of *Sik1* in the PVN neurons, we utilized an adeno-associated virus 2/9 (AAV2/9), a well-characterized serotype known for efficient neuronal transduction in the central nervous system [29, 30], under the control of the neuron-specific human synapsin (hSyn) promoter [31]. Specifically, *Sik1*^flox/flox^ mice were injected with either AAV2/9–hSyn–EGFP (PT-1990, BrainVTA Co., Ltd.) or AAV2/9–hSyn–Cre–EGFP (PT-1168, BrainVTA Co., Ltd.), respectively. Briefly, 8-week-old *Sik1*^flox/flox^ mice were deeply anesthetized with isoflurane via inhalation and placed on a stereotaxic apparatus. Each virus (150 nL, 50 nL/min, 10^12^ viral particles/mL) was infused into the PVN region using the following stereotaxic coordinates: Anterior-posterior, −0.8 mm; Medial-lateral, ±0.30 mm; Dorsal-ventral, −4.8 mm relative to bregma. The injection microelectrode was slowly withdrawn over 10 min to ensure proper virus diffusion. After a 3-week recovery period, at least three mice were sacrificed for immunofluorescence analysis to assess the efficiency of viral transfection and Cre expression, and the other mice were used for subsequent experiments.

### Acute Brain Slice Preparation and Cultures

Animals (postnatal day 21) were anesthetized via isoflurane inhalation and subsequently decapitated. Their brains were rapidly dissected and immersed in ice-cold sucrose-based artificial cerebrospinal fluid (aCSF) containing the following components (in mM): 210 sucrose, 2.5 KCl, 1 NaH_2_PO_4_, 26 NaHCO_3_, 7 MgSO_4_, 0.5 CaCl_2_, and 7 dextrose. Coronal brain slices (300 µm thickness) were sectioned using a vibratome and immediately transferred to a holding chamber containing either normal aCSF or high-salt aCSF, respectively, where they were incubated for 3 h at 34 °C. The composition of normal aCSF was as follows (in mM): 120 NaCl, 2.5 KCl, 1 NaH_2_PO_4_, 1.3 MgSO_4_, 2.5 CaCl_2_, and 26.2 NaHCO_3_. The composition of the high-salt aCSF was identical to that of the normal aCSF except for the elevated NaCl concentration (170 mM). Subsequently, the slices were postfixed in 4% PFA for 10–12 h at 4 °C and transferred to 15% and 30% sucrose in phosphate buffer for 10–12 h at 4 °C.

### Single-Nucleus RNA Sequencing

#### Tissue Dissociation

According to the manufacturer’s instructions, single-nucleus RNA sequencing was performed on the PVN micro-dissected from the following groups: wild-type mice fed with an NSD, *Sik1*^–/–^ mice fed with an NSD, wild-type mice fed with an HSD, and *Sik1*^–/–^ mice fed with an HSD. As previously described [32], the PVN tissue was obtained as follows. Mice were transcardially perfused with PBS following deep anesthesia induced by isoflurane. The brains were promptly dissected and maintained in oxygenated, ice-cold aCSF during the dissection process. Coronal Sects. (0.5 mm thickness) encompassing the extended PVN (approximately −0.58 mm to −1.22 mm from Bregma) were prepared using an adult mouse brain matrix and a double-edge razor blade. The PVN issue was then meticulously micro-dissected from each brain section under a stereomicroscope. The PVN tissue samples from nine mice per group were pooled, followed by lysis and resuspension to obtain a single-nucleus suspension.

#### Single-Nucleus Library Preparation and Sequencing

Single-nucleus suspensions from the PVN were loaded onto a 10 × Genomics Chromium chip. Reverse transcription and library preparation were performed using the Single Cell 3' Reagent Kit v2 (10 × Genomics, Cat. #PN-120237). The constructed libraries were pooled and subjected to sequencing on an Illumina NovaSeq platform using a PE150 Xp workflow, generating 150-bp paired-end reads with an average sequencing depth exceeding 30 ×.

#### Quality Control and Identification of Cell Clusters

Data preprocessing was performed using the 10 × Genomics Cell Ranger software (version 2.1.1) in default mode. The reference data supplied by 10 × Genomics for the mm10 assembly, along with corresponding gene annotations, was used for alignment and quantification. Subsequent analyses were conducted using the Bai Yin Yun Website platform (version 3.0) (http://www.bioinforcloud.org.cn). After applying quality control filters based on gene counts (1,000–3,000), UMI counts (0–20,000), and mitochondrial gene fraction (> 0.05), a total of 322,815 cells were retained for further analysis. Batch effects originating from chromium channels were removed from normalized data using the RunSeurat tool. For cell clustering analysis, we applied the UMAP method with the spread parameter set to 60 and the resolution parameter set to 3.22811, incorporating cell type-specific marker genes. Finally, cell types were determined by integrating marker genes from literature [33–37] with gene ontology annotations using the analytical tools available on the Bai Yin Yun Website platform.

#### Image Analysis and Quantification

For analyzing microglia morphology, brain sections containing the hypothalamic PVN were selected for immunohistochemical staining with the microglial marker Iba1. For each animal, three consecutive sections spanning the PVN were imaged using a high-resolution microscope. From each section, 15–20 Iba1-positive microglia were randomly selected within a predefined area of 40,000 μm^2^ in the PVN region. Microglial morphology was traced and digitized using the Neurolucida software system (MBF Bioscience). The following parameters were quantitatively analyzed: soma size, process length, number of branches. Sholl analysis was further performed to evaluate process complexity. Concentric circles were drawn at radial intervals of 10 μm from the soma centroid. The number of intersections between microglial processes and each circle was quantified to generate Sholl profiles for individual cells. Group-wise Sholl curves were compared to assess differences in process complexity.

To evaluate the expression levels of Sik1, IL-1β, and phosphorylated p65 in the PVN using immunofluorescence staining, we performed quantitative analysis of fluorescence intensity. For each mouse, 2–3 consecutive brain sections containing the PVN were imaged under consistent microscopic parameters. The PVN region was precisely delineated as the region of interest (ROI) using ImageJ software, and the mean fluorescence intensity within this ROI was measured for each section after background subtraction. The values from multiple sections per animal were averaged to obtain a representative value for each mouse.

For the assessment of Sik1 protein expression levels in the PVN using IHC with DAB staining, we employed integrated optical density (IOD) measurement. Following the same section selection criteria, the PVN was identified and outlined as ROI in each section. The IOD value, which represents the total staining intensity within the ROI, was measured using ImageJ's optical density analysis tools. Measurements from 2–3 sections per animal were similarly averaged to generate a representative value.

#### Quantification and Statistical Analysis

The investigators were blinded to the genotypes and treatments of the animals. The Shapiro–Wilk test was used to assess the normality of the data. For normally distributed data, Student’s *t-*test was applied; non-parametric tests were used for non-normally distributed data or small sample sizes. One-way ANOVA with *post hoc* Dunnett’s tests was employed for comparisons between multiple groups. To analyze the effects of two different factors on a continuous variable, two-way repeated measures ANOVA with *post hoc* Bonferroni tests was performed. Data mapping was performed using Adobe Photoshop and GraphPad Prism Version 9.0, while statistical analysis was conducted using SPSS 26.0. Data are presented as mean ± SEM, and statistical significance was set at *P* < 0.05.

## Results

### High-Salt Diet Increases Sik1 Expression in the Hypothalamic PVN

**Fig. 1:**
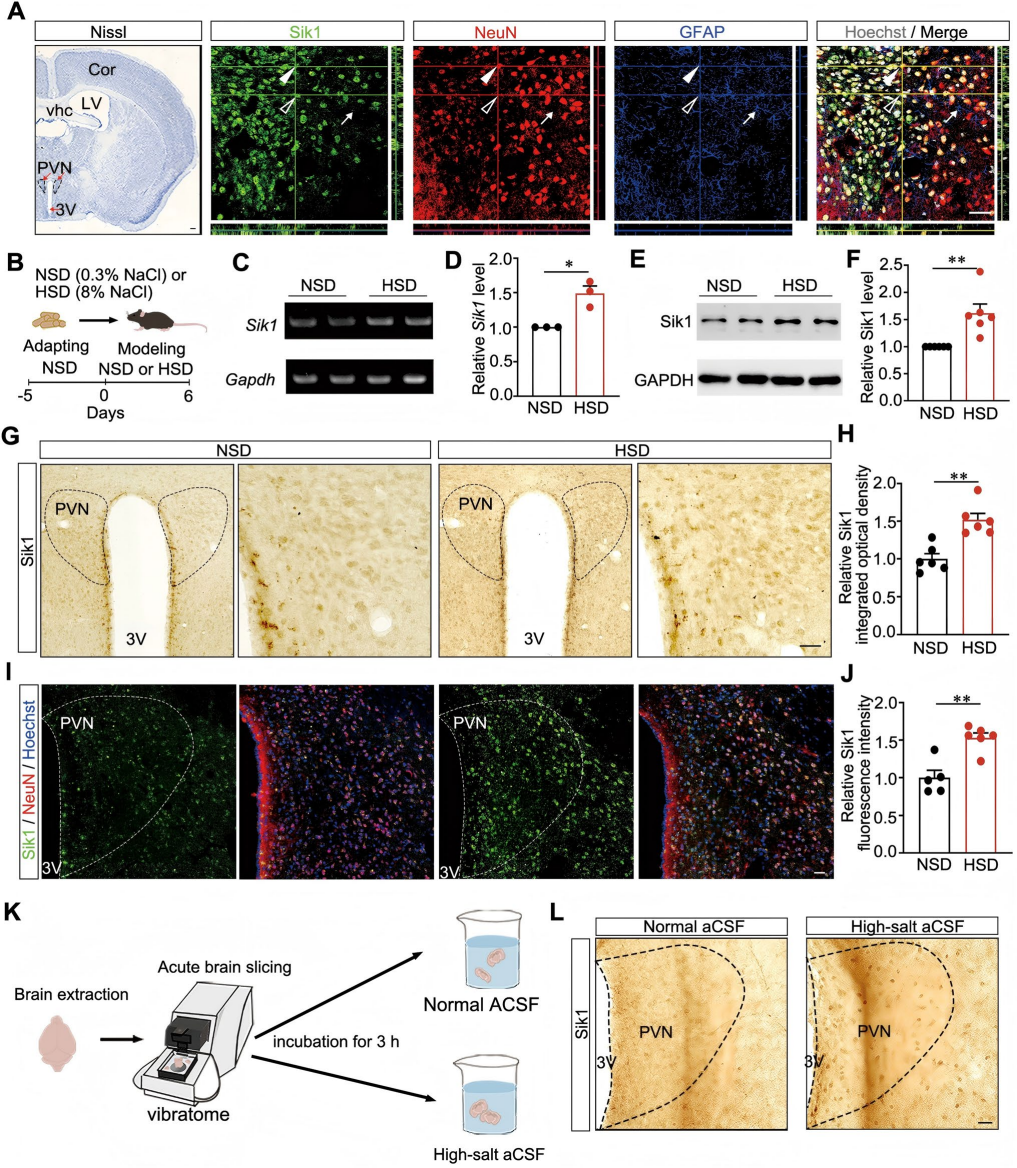
The expression of Sik1 in the PVN was increased following high-salt treatment. (**A**) The distribution of Sik1 protein in the PVN of mice. Leftmost panel: Nissl staining of coronal brain sections of mice; Scale bar, 200 μm. Rightmost four panels: Magnified z-stack images of the distribution of Sik1 in the PVN detected by immunostaining. NeuN and GFAP were used as protein markers for neurons or glial cells, respectively. Hoechst was used for counterstaining of the nucleus (gray). Arrows and empty arrows indicate NeuN+ cells and NeuN– cells, respectively; arrowheads indicate GFAP+ cells; Scale bar, 30 μm. (**B**) Schematic of the HSD or NSD feeding protocol in mice. (**C**–**D**) PCR analysis of *Sik1* mRNA expression in the hypothalamus after high salt intake. (**C**) Representative bands of RT-PCR experiments. (**D**) Semi-quantitative results of real-time PCR experiments. *Gapdh* was used as an internal control. *n* = 3 samples *per* group. (**E**–**F**) Western blotting analysis of the indicated proteins in the hypothalamus after high salt intake. GAPDH immunoblotting verified equal. (**E**) Representative blots. (**F**) Statistical results. *n* = 6 samples *per* group. (**G**) DAB staining showed that Sik1 protein expression increased in the PVN after HSD. Scale bar, 30 μm. (**H**) Quantification of the integrated optical density (IOD) of Sik1 in the PVN. *n* = 6 mice *per* group, ​**P < 0.01. (**I**) Immunostaining showed that Sik1 protein expression was increased in the PVN after HSD. (**J**)​​ Quantification of the fluorescence intensity of Sik1 in the PVN. NSD group, *n* = 5 mice, HSD group, *n* = 6 mice. (**K**) Schematic diagram of acute brain slices’ incubation with high [Na^+^] in vitro. (**L**) DAB staining showed that Sik1 protein expression increased in the PVN after culturing in high [Na^+^] aCSF. Scale bar, 30 μm. *P < 0.05, **P < 0.01. Two-tailed unpaired Student’s *t*-test. Data are expressed as mean ± SEM. Cor, cortex; vhc, ventral hippocampal commissure; LV, lateral ventricle; PVN, hypothalamic paraventricular nucleus; 3V, third ventricle

Firstly, we performed immunohistostaining detection to observe the expression of Sik1 in the hypothalamic PVN, a key brain region for regulating blood pressure. By co-immunostaining with multiple protein markers, results showed that Sik1 protein was expressed by NeuN-positive neurons, but not in GFAP-positive astrocytes or Iba1-positve microglia (Fig. 1A and Fig. S1A and B). To confirm whether neuronal Sik1 is sensitive to high salt in this area, we used reverse transcription polymerase chain reaction (RT-PCR) and real-time PCR to detect the expression level of *Sik1* mRNA in mice fed with an acute HSD (Fig. 1B). We observed an increase in the *Sik1* mRNA expression level of 1.49 ± 0.11 fold above the control level in the hypothalamus after HSD feeding (*P* = 0.01, Fig. 1C and D). Subsequently, we determined the expression of Sik1 protein in the hypothalamus after HSD feeding, finding a 1.62 ± 0.17 fold increase within the hypothalamus of mice after high salt intake (*P* = 0.004, Fig. 1E and F). ​To determine the spatial distribution of upregulated Sik1, we performed immunohistochemical analysis and found that Sik1 was increased within the PVN with 1.52 ± 0.09 fold change after HSD (*P* = 0.001, Fig. 1G and H). Immunofluorescence staining further confirmed the increase of Sik1 expression, revealed by fluorescence intensity, within the PVN of the HSD group (*P* = 0.001, Fig. 1I and J). Furthermore, to avoid the confounding effects of high salt in vivo and examine its direct effect on neuronal Sik1,we developed an in vitro model using acute brain slices incubated directly in high-salt aCSF (Fig. 1K). Remarkably, we found that Sik1 expression significantly increased in the PVN of slices incubated in high-salt aCSF (Fig. 1L). Taken together, these results showed that Sik1 in PVN neurons is responsive to systemic high-salt intake.

### Ablation of Sik1 Results in BP Elevation Upon HSD

**Fig. 2:**
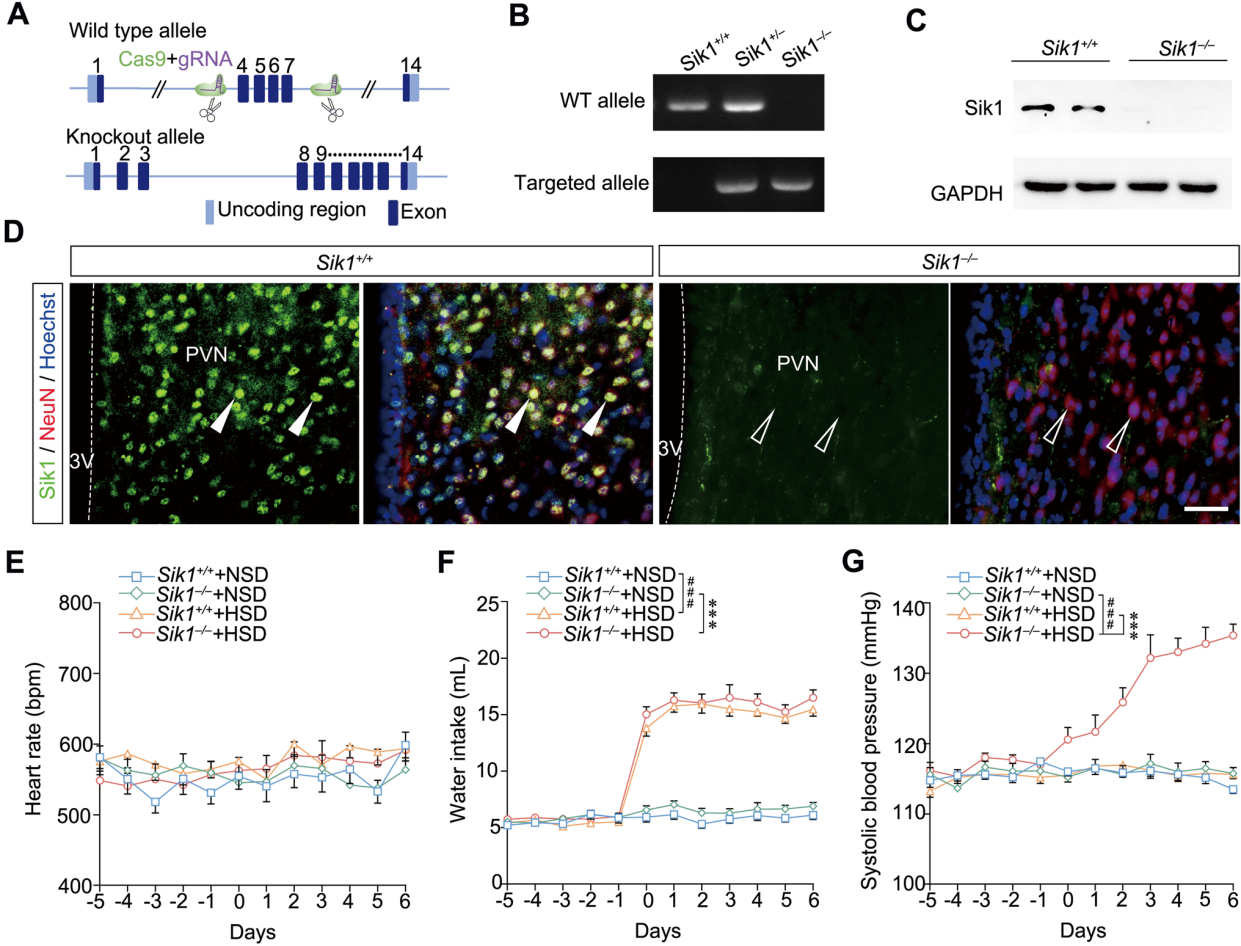
*Sik1*-mutant mice showed increased blood pressure following an HSD. (**A**) Schematic diagrams for the generation of *Sik1*^−/−^ mice. (**B**) Genotyping of *Sik1*^−/−^ mice. (**C**) Representative blots of Sik1 expression in mouse hypothalamus. (**D**) Immunofluorescence staining showed no detection of Sik1 protein in the hypothalamus in *Sik1*^–/–^ mice. Arrows and empty arrows indicate Sik1+ cells and Sik1– cells, respectively. Scale bar, 30 μm. (**E**) No significant difference in heart rate between *Sik1*^–/–^ and *Sik1*^+/+^ mice on either an HSD or an NSD. (**F**) Increased water intake in *Sik1*^–/–^ mice fed an HSD. ***P < 0.001, *Sik1*^−/−^ + HSD *vs Sik1*^−/−^ + NSD, ^###^*P* < 0.001, *Sik1*^+/+^  + HSD *vs Sik1*^+/+^  + NSD. (**G**) Increased blood pressure in *Sik1*^–/–^ mice upon high salt intake. ***P < 0.001, *Sik1*^−/−^ + HSD *vs Sik1*^+/+^ + HSD; ^###^ *P* < 0.001, *Sik1*^−/−^ + HSD *vs Sik1*^−/−^  + NSD. *n* = 18 mice *per* group. Two-way ANOVA with *post hoc* Bonferroni test. Data are expressed as mean ± SEM

To determine whether Sik1 is involved in regulating blood pressure, we performed a series of experiments in *Sik1* knockout (KO) mice with global ablation of Sik1 (Fig. 2A and B). Both western-blot analysis and immunofluorescence staining further confirmed the efficacy of the knockout of the *Sik1* gene in mice (Fig. 2C and D). Subsequently, we fed *Sik1*^–/–^ mice and their wild-type littermates an 8% NaCl diet following the high-salt dietary modeling method. First, no significant differences in heart rate were observed between the two genotypes, whether they were fed an NSD or an HSD (Fig. 2E). Meanwhile, both *Sik1*^–/–^ mice and their littermate controls fed with an HSD exhibited increased water intake [*P* < 0.001, *Sik1*^−/−^ + HSD *versus* (*vs*) *Sik1*^−/−^ + NSD; *P* < 0.001, *Sik1*^+/+^  + HSD *vs Sik1*^+/+^  + NSD, Fig. 2F]. Subsequently, we observed no significant difference in SBP between the two genotypes following NSD intake (Fig. 2G). Notably, after three days on an HSD, *Sik1*^–/–^ mice exhibited elevated SBP and continued to rise during the following 4–7 days, compared to their wild-type littermates (*P* < 0.001, *Sik1*^−/−^ + HSD *vs Sik1*^+/+^ + HSD; *P* < 0.001, *Sik1*^−/−^  + HSD *vs Sik1*^−/−^  + NSD, Fig. 2G). These results showed that the BP increased in *Sik1-*KO mice following an acute HSD, indicating the potential role of Sik1 in preventing BP elevation.

### Sik1 in PVN Neurons Prevents BP Elevation by HSD

**Fig. 3:**
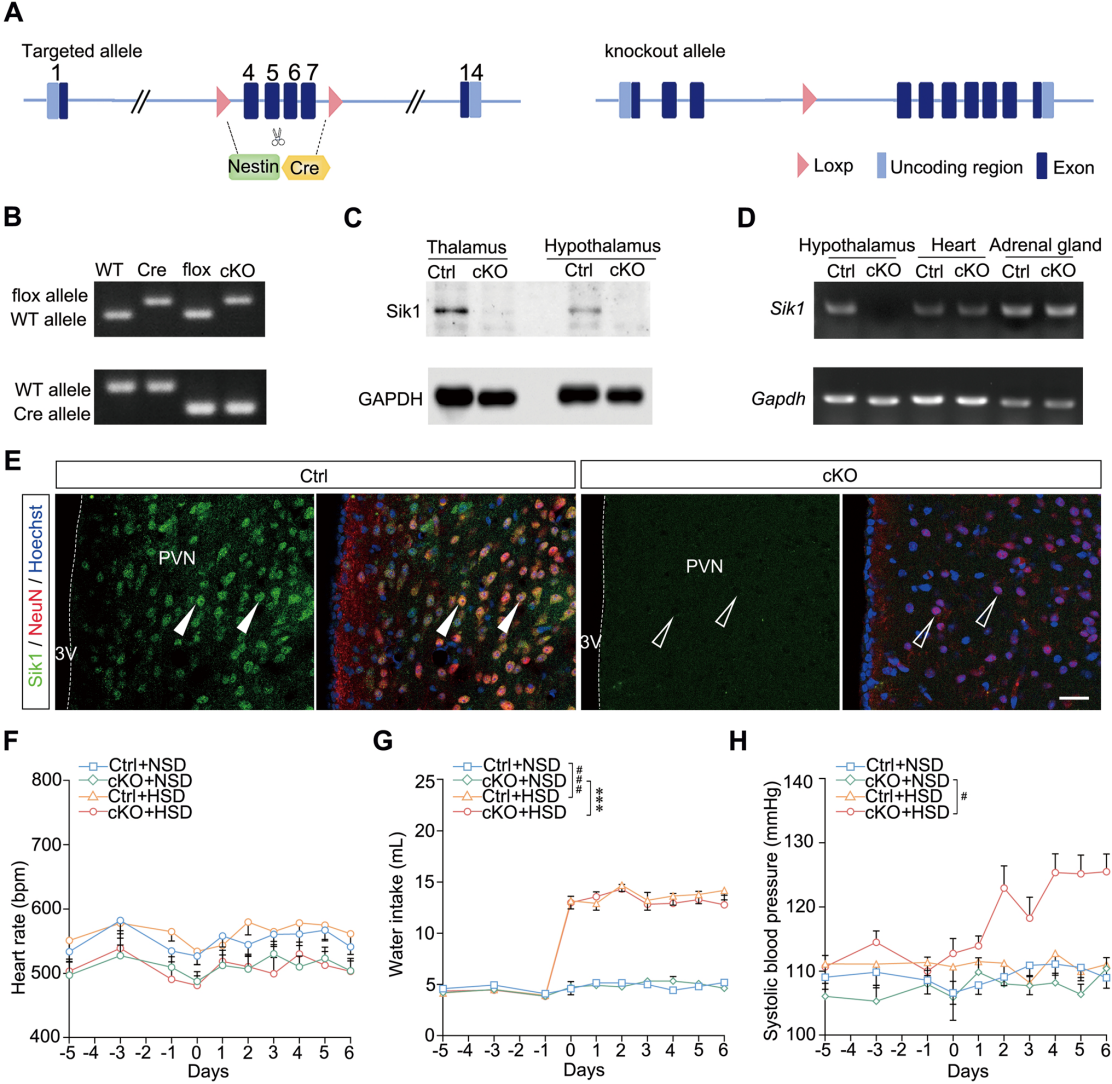
Sik1 ablation in neurons led to increased blood pressure following an HSD. (**A**) Schematic diagram for the generation of *Nestin-Cre;Sik1*^−/−^ mice. (**B**) Genotyping of *Nestin-Cre;Sik1*^−/−^ mice. (**C**) Western blotting analysis of Sik1 expression in the brain of *Nestin-Cre; Sik1*^−/−^ mice and their control littermates. (**D**) RT-PCR of *Sik1* expression in peripheral tissue of *Nestin-Cre;Sik1*^−/−^ mice. (**E**) Immunofluorescence staining showed no detection of Sik1 protein in the hypothalamus in *Nesin-Cre;Sik1*^–/–^ mice. Arrows and empty arrows indicate Sik1+ cells and Sik1– cells, respectively. Scale bar, 30 μm. (**F**) No significant difference in heart rate between cKO and their littermates after either HSD or NSD. (**G**) Increased water intake in *Nestin-Cre;Sik1*^−/−^ mice fed with an HSD. ***P < 0.001, cKO + HSD *vs* cKO + NSD; ^###^*P* < 0.001, Ctrl + HSD *vs* Ctrl + NSD. (**H**) Increased blood pressure in *Nestin-Cre;Sik1*^−/−^ mice upon high salt intake. ^#^*P* < 0.05, cKO + HSD *vs* cKO + NSD. *n* = 15 mice *per* group. Two-way ANOVA with *post hoc* Bonferroni test. Data are expressed as mean ± SEM.

To explore Sik1’s specific role in the brain with respect to BP during HSD feeding, we generated *Nestin-Cre;Sik1*^*–/–*^ mice, which selectively lack Sik1 in the nervous system [26, 27] (Fig. 3A and B). Both western-blot analysis and RT-PCR confirmed the knockout efficacy of *Sik1* in the brain, but not in peripheral tissues (Fig. 3C and D). Immunostaining further showed that Sik1 was deletion in PVN neurons (Fig. 3E). We detected no significant differences in the heart rate of mice on an HSD, compared with that of mice fed an NSD (Fig. 3F). *Nestin-Cre;Sik1*^*–/–*^ mice, upon high salt intake, exhibited significantly increased water intake, compared with that observed in both *Nestin-Cre;Sik1*^*–/–*^and control mice fed with an NSD (*P* = 0.001, cKO + HSD *vs* cKO + NSD; *P* = 0.004, Ctrl + HSD *vs* Ctrl + NSD, Fig. 3G). After 3 days on the HSD, the SBP of *Nestin-Cre;Sik1*^*–/–*^ mice was higher than that of cKO mice fed with an NSD. By day 7^th^ of the HSD, the SBP of *Nestin-Cre;Sik1*^*–/–*^ mice had further increased (*P* = 0.031, cKO + HSD *vs* cKO + NSD, Fig. 3H).

**Fig. 4:**
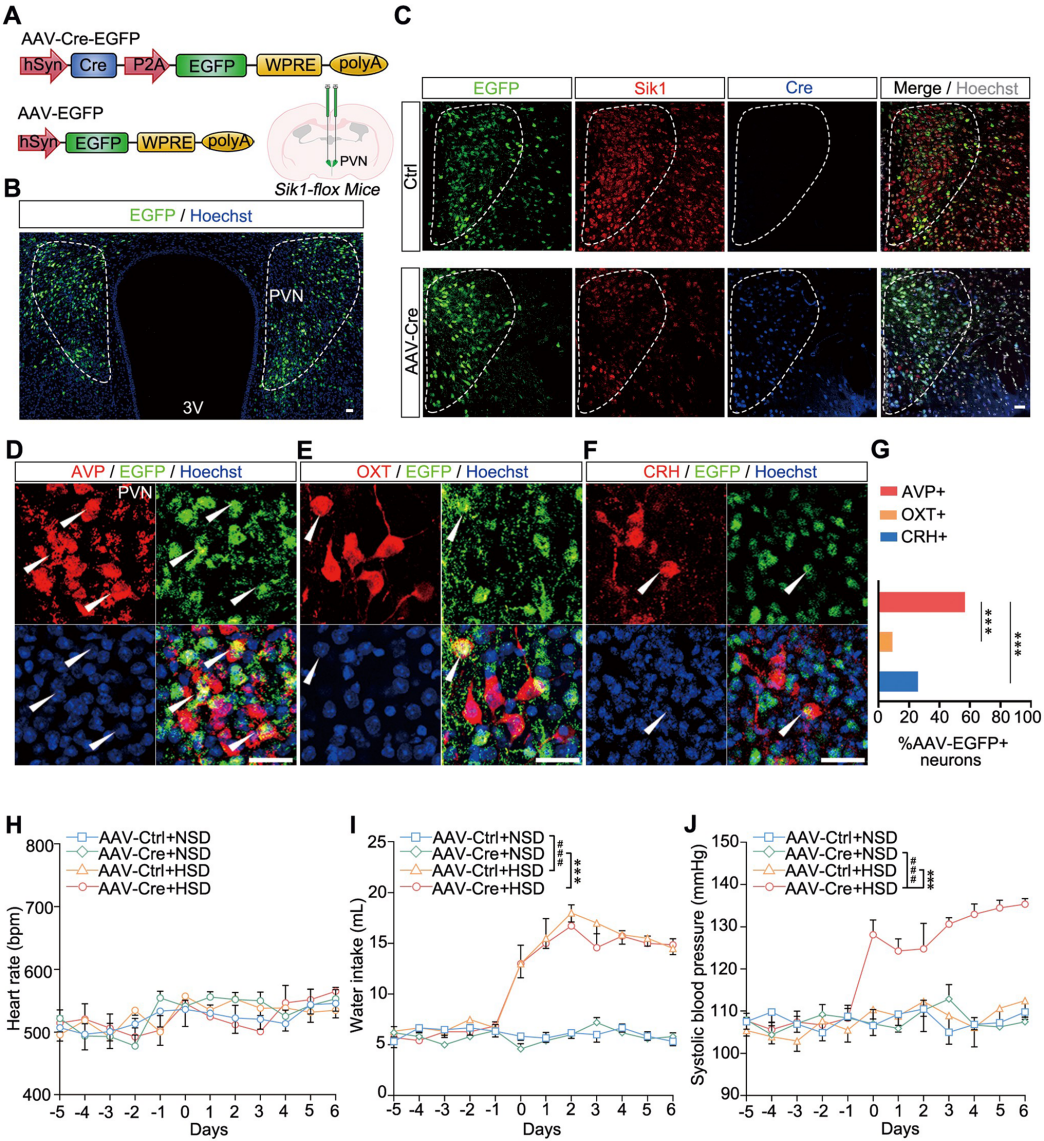
AAV-mediated deletion of *Sik1* in the PVN neurons induces elevated blood pressure following an HSD. (**A**) Schematic diagram of AAV construct and injection into the PVN of mice. AAV-Cre-EGFP or control AAV-EGFP was bilaterally injected into the PVN of 6- to 8-week-old *Sik1*^flox/flox^ mice. (**B**) Immunofluorescence of bilateral infection of the PVN by AAV-hSyn-EGFP. Scale bar, 30 μm (**C**) Immunostaining results showing that AAV-mediated Cre expression ablated Sik1 protein in the infected neurons. Scale bar, 30 μm. (**D**)​​ Immunofluorescence  detection for EGFP (green) and AVP (red) shows co-localization of the AAV-hSyn-EGFP vector with AVP-positive neurons in the PVN. Arrows indicate double-labeled neurons. Scale bar, 30 μm. ​​(**E**)​​ Immunofluorescence detection for EGFP (green) and OXT (red) shows little co-localization of the AAV-hSyn-EGFP vector with OXT-positive neurons in the PVN. Arrows indicate double-labeled neurons. Scale bar, 30 μm.​​ (**F**)​​ Immunofluorescence detection for EGFP (green) and CRH (red) shows little co-localization of the AAV-hSyn-EGFP vector with CRH-positive neurons in the PVN. Arrows indicate double-labeled neurons. Scale bar, 30 μm.​​ (**G**) Statistical results showed the percentage of AVP-positive, OXT-positive, and CRH-positive neurons containing AAV-EGFP signals, respectively. AVP-positive neurons group, *n* = 4 mice; OXT-positive neurons group, *n* = 3 mice; CRH-positive neurons group, *n* = 3 mice. ***P < 0.001, Chi-square test with post hoc Bonferroni test. (**H**) No significant difference in heart rate between *AAV-Cre;Sik1*^−/−^ and their littermates after either HSD or NSD. (**I**) Increased water intake in *AAV-Cre; Sik1*^−/−^ mice fed an HSD. ***P < 0.001, AAV-Cre + HSD *vs* AAV-Cre + NSD; ^###^*P* < 0.001, AAV-Ctrl + HSD *vs* AAV-Ctrl + NSD. (**J**) Increased blood pressure in *AAV-Cre;Sik1*^−/−^ mice upon high salt intake. ***P < 0.001, AAV-Cre + HSD *vs* AAV-Ctrl + HSD; ^###^*P* < 0.001, AAV-Cre + HSD *vs* AAV-Cre + NSD. *n* = 6 mice *per* group. Two-way ANOVA with *post hoc* Bonferroni test. Data are expressed as mean ± SEM

To further confirm the high expression of Sik1 in the PVN and its critical role in BP regulation, we bilaterally injected AAV2/9–hSyn–Cre–EGFP into the PVN of *Sik1*^flox/flox^ mice (Fig. 4A and B). Compared with the control group, immunostaining analysis showed that AAV2/9–hSyn–Cre–EGFP injection resulted in selective ablation of Sik1 expression exclusively within the infected neurons (Fig. 4C). To analyze which cell types recieved this AAV infection, we further performed immunofluorescent co-staining. The results showed that high infection rates in AVP-positive neurons (56.68%) and CRH-positive neurons (25.93%), whereas infection of OXT-positive neurons was minimal (9.09%) (Fig. 4D–G). Subsequently, we assessed the physiological consequences of Sik1 deletion in the PVN under either NSD or HSD feeding conditions. There was no significant difference in heart rate between *AAV-Cre*;*Sik1*^*–/–*^ mice and their controls under either NSD or HSD feeding conditions (Fig. 4H). Additionally, we observed a significant increase in water intake in both *AAV-Cre; Sik1*^–/–^ mice and their control mice on an HSD (*P* < 0.001, AAV-Cre + HSD *vs* AAV-Cre + NSD; *P* < 0.001, AAV-Ctrl + HSD *vs* AAV-Ctrl + NSD, Fig. 4I). Importantly, SBP increased in those mice with AAV-Cre-mediated Sik1 deletion fed with an HSD (*P* < 0.001, AAV-Cre + HSD *vs* AAV-Ctrl + HSD; *P* < 0.001, AAV-Cre + HSD *vs* AAV-Cre + NSD, Fig. 4J). Thus, these findings suggest that Sik1 deletion in PVN neurons is sufficient to induce blood pressure elevation upon a high-salt diet.

### Sik1 in AVP-Positive Neurons Attenuates HSD-Induced BP Elevation via NF-κB Signaling Pathway

**Fig. 5:**
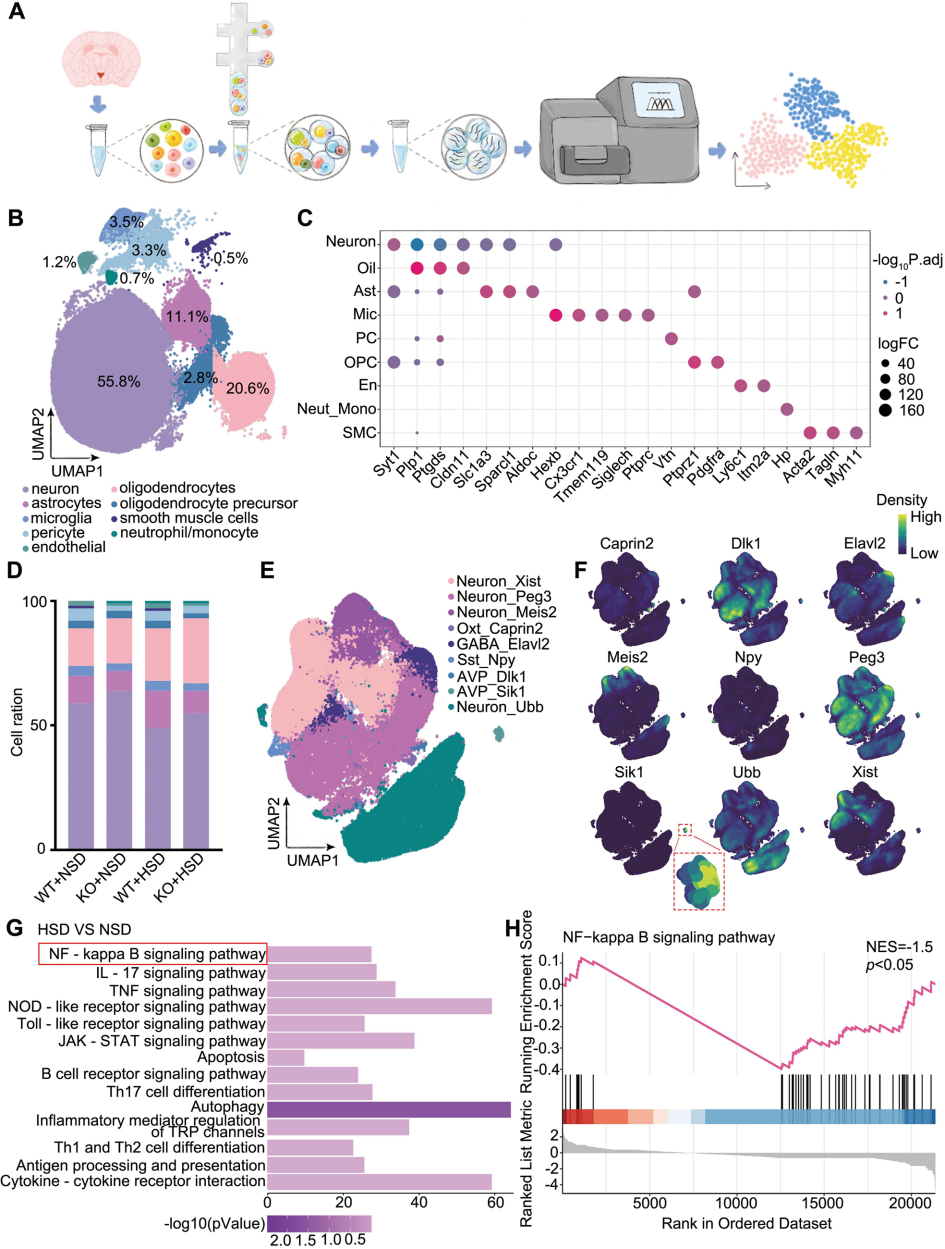
Single-cell RNA sequencing of cells in the PVN of WT and KO mice under NSD or HSD conditions. (**A**) Flowchart. (**B**) Mapping of various cell subpopulations in the single-cell atlas. (**C**) Bubble chart showing cell type annotating genes. (**D**) Stacked bar chart showing differences in cell abundance of different groups. (**E**) UMAP projection highlighting the cellular cluster characterized by high Sik1expression. (**F**) Density plot showing the markers of PVN neurons subpopulations. (**G**) KEGG pathway enrichment analysis of differentially expressed genes in the ​Sik1-positive AVP neuronal subpopulation under HSD conditions.​​ (**H**)​​ Gene Set Enrichment Analysis (GSEA) plot for NF-κB signaling showing the enrichment score profile across the rank-ordered gene list

To further explore the cellular and molecular mechanisms underlying the effect of Sik1 on blood pressure under high salt conditions, we utilized single-nucleus RNA sequencing technology for high-throughput analysis of the PVN samples (Fig. 5A). After quality control, 61,413 cells from wild-type mice fed with an NSD, 69,308 cells from *Sik1*^–/–^ mice fed with an NSD, 105,073 cells from wild-type mice fed an HSD, and 87,017 cells from *Sik1*^–/–^ mice fed an HSD mice were analyzed jointly. Based on their unique gene markers, we identified nine distinct cell types, including neurons, oligodendrocytes, astrocytes, microglia, pericyte, oligodendrocyte precursor cells, endothelial, neutrophil/monocyte, and smooth muscle cells (Fig. 5B and C). The neurons constituted the predominant populations, followed by oligodendrocytes, astrocytes, and microglia across all groups (Fig. 5D). We then performed further analysis on Sik1 expression within neuronal subpopulations and found that Sik1 may be primarily expressed by AVP-positive neurons (Fig. 5E and F). Subsequently, we performed KEGG enrichment analysis on the differentially expressed genes in the Sik1-expressing within AVP neuronal subpopulation under HSD conditions, compared to NSD conditions. We found that involvement of several signaling pathways, of which NF-κB signaling pathway inhibited after an HSD (normalized enrichment score, NES = −1.5; *P* < 0.05; Fig. 5G and H).

**Fig. 6:**
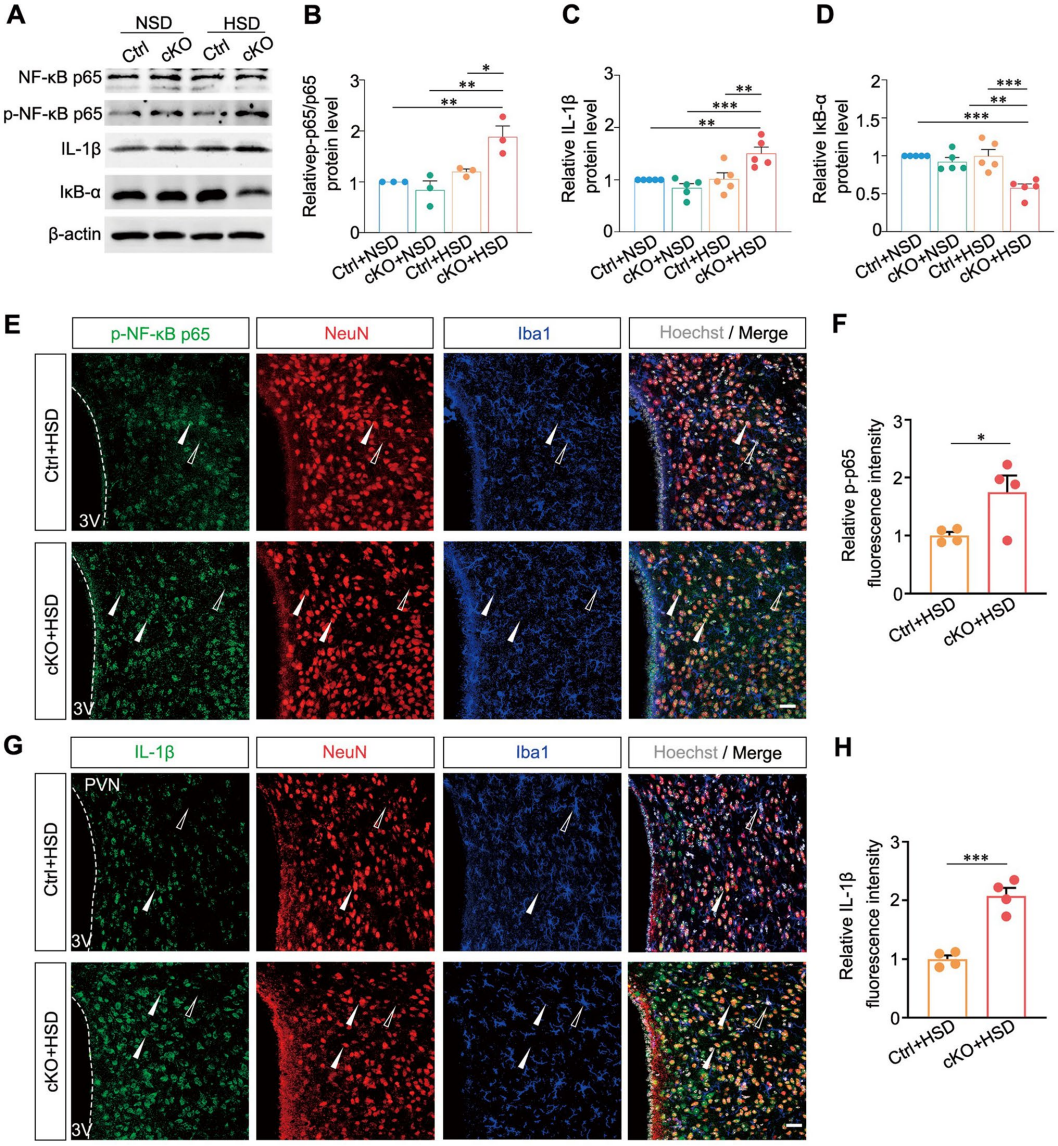
Changes of neuronal NF-κB p65 signaling pathway in the absence of Sik1 upon high salt intake. (**A**–**D**). Representative immunoblot bands and quantitative analysis showed the expression of IĸB-α, NF-κB p65, p-NF-κB p65 and IL-1β in the hypothalamus between the four groups. β-actin immunoblotting verified equal loading. **A**, Representative western blots; **B**, Statistical results of IĸB-α, *n* = 5 mice *per* group; **C**, Statistical results of the ratio of p-NF-κB p65 and NF-κB p65, *n* = 3 mice *per* group; **D**, Statistical results of IL-1β, *n* = 5 mice *per* group. One-way ANOVA with *post hoc* Dunnett’s test. (**E**) Immunofluorescence staining showing increased p-p65 (green) expression in cKO mice on an HSD. NeuN (Red) and Iba1 (blue) were used as markers for neurons and microglia, respectively. Arrows indicate NeuN-positive cells, while empty arrows indicate Iba1-positive cells. Scale bar, 30 μm. (**F**) Quantification of the fluorescence intensity of p-p65 in the PVN. (**G**) Immunofluorescence staining showing increased IL-1β (green) in cKO mice on an HSD. Scale bar, 30 μm. (**H**) Quantification of the fluorescence intensity of IL-1β in the PVN. Ctrl + HSD group, *n* = 4 mice; cKO + HSD group, *n* = 4 mice. *P < 0.05, **P < 0.01, ***P < 0.001. Two-tailed unpaired Student’s t-test. Data are expressed as mean ± SEM

As NF-κB p65-driven neuroinflammation in the pathogenesis of hypertension [38, 39], we next used biochemical experiments to observe the expression level of NF-κB p65 pathway in *Sik1*-cKO mice after an HSD. Results revealed phosphorylated NF-κB p65 (p-p65) was significantly increased by approximately 1.9-fold​ compared with the Ctrl + NSD group (*P* = 0.005), ​2.2-fold​ compared with the cKO + NSD group (*P* = 0.002), and ​1.6-fold​ compared with the Ctrl + HSD group (*P* = 0.021) (Fig. 6A and B). Subsequently, we examined the expression of IL-1β, a key pro-inflammatory cytokine transcriptionally regulated by NF-κB. Its level was significantly upregulated in the PVN of *Sik1*-cKO mice under HSD conditions, showing an increase of approximately 1.5-fold compared with Ctrl + NSD group (*P* = 0.004), 1.8-fold compared with cKO + NSD mice (*P* < 0.001), and 1.5-fold compared with Ctrl + HSD mice (*P* = 0.005) (Fig. 6A and C). Furthermore, immunostaining results showed that elevated levels of both p-p65 (*P* = 0.044, Fig. 6E and F) and IL-1β (*P* < 0.001, Fig. 6G and H) were predominantly localized within NeuN-positive cells in the PVN. Taken above, these results imply that presence of Sik1 leads to inhibition of neuron-centered NF-κB p65 activation in PVN neurons.

### Microglia are Activated in the PVN Lacking Sik1 After an HSD

**Fig. 7:**
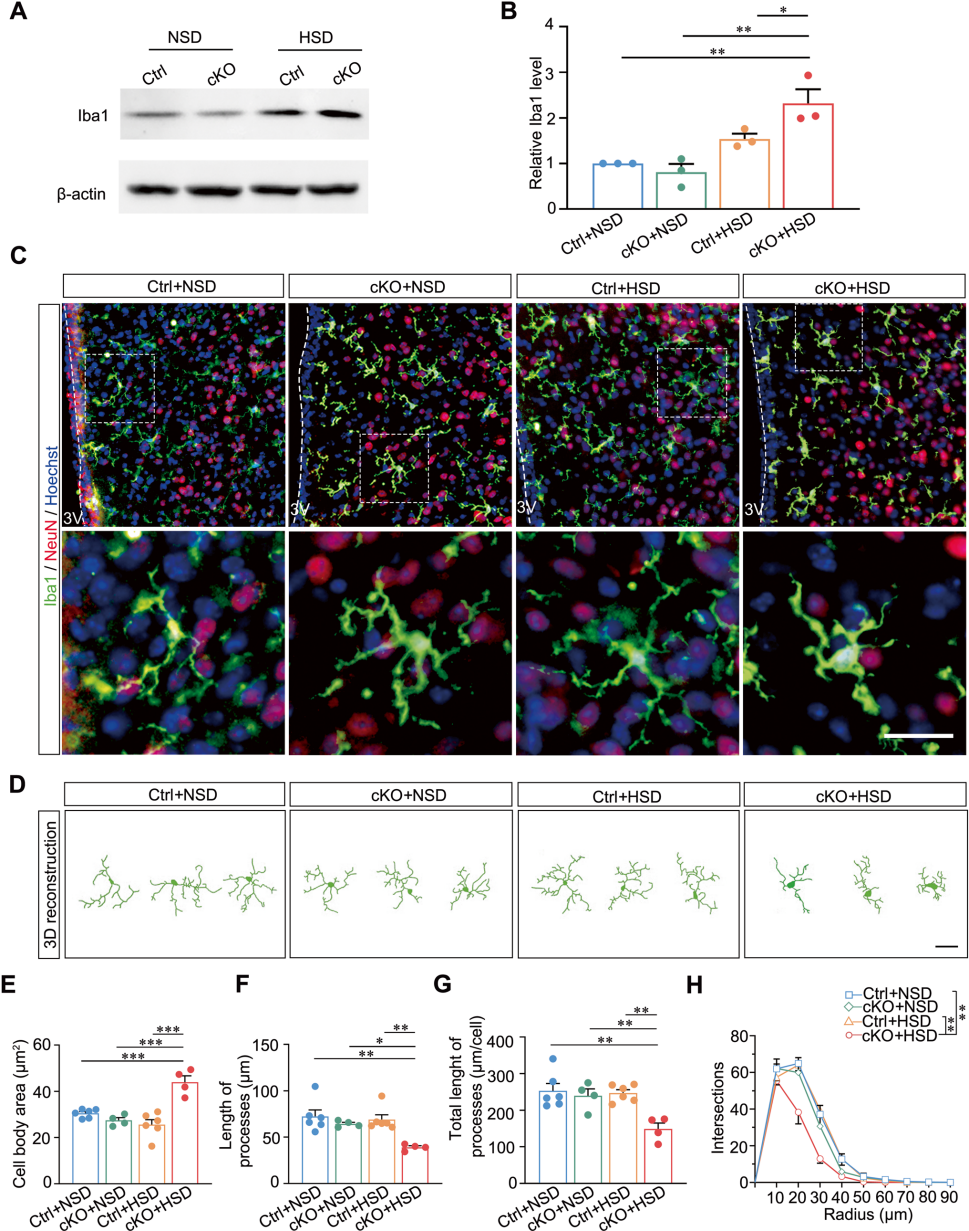
Activation of the microglia within the PVN of *Nestin-Cre; Sik1*^–^/^–^ mice following an HSD. (**A – B**) Representative immunoblot blots and quantitative analysis showed the expression of Iba1 in the hypothalamus between the four groups. β-actin immunoblotting verified equal. **A**, Representative western blots; **B**, Statistical results. (**C**) Representative images of Iba1 immunofluorescence in the mouse PVN. Boxed frames indicate microglia. Scale bar, 30 μm. (**D**) Neurolucida tracings of representative Iba1-immunolabeled microglia. Scale bar, 30 μm. (**E**) Statistical results of body area of microglia within the PVN. (**F**) Statistical results for the average length of microglial processes. (**G**) Statistical results for the total length of processes *per* microglia. One-way ANOVA with *post hoc* Dunnett’s test. (**H**) Sholl analysis was performed to quantify the number of intersections at increasing radial distances from the microglial soma within the PVN. Two-way ANOVA with *post hoc* Bonferroni test. Ctrl + NSD, *n* = 6; cKO + NSD, *n* = 4; Ctrl + HSD, *n* = 6; cKO + HSD, *n* = 4. At least 15 of the largest microglial cells in the 40,000 μm^2^ hypothalamic area were counted for each mouse. *P < 0.05, **P < 0.01, ***P < 0.001. Data are expressed as mean ± SEM

Given that NF-κB-mediated neuroinflammation can activate microglia [40], next we observed whether microglia activation occured in *Nestin-Cre;Sik1*^–/–^ mouse PVN. Indeed, we detected an increase in the expression level of Iba1 protein, an indicator for microglia activation, in the PVN of mutant mice and their control littermates, both of which were fed with an HSD. ​We found that Iba1 expression levels were significantly elevated by approximately 2.3-fold compared with Ctrl + NSD group (*P* = 0.003), 2.9-fold compared with cKO + NSD mice (*P* = 0.001), and 1.5-fold compared with Ctrl + HSD mice (*P* = 0.044) (Fig. 7A and B). Furthermore, we performed morphology analysis of microglial combined with immunostaining and 3D reconstruction by Neurolucida software (Fig. 7C and D). We observed that the average soma size was significantly increased by approximately 1.44-fold compared with Ctrl + NSD (43.94 ± 2.75 µm^2^ *vs* 30.48 ± 0.77 µm^2^, *P* < 0.001), 1.60-fold compared with cKO + NSD (43.94 ± 2.75 µm^2^ *vs* 27.45 ± 1.18 µm^2^, *P* < 0.001), and 1.72-fold compared with Ctrl + HSD (43.94 ± 2.75 µm^2^ *vs* 25.61 ± 2.13 µm^2^, *P* < 0.001) (Fig. 7E). In contrast, notable reductions were observed in the microglial process length, which was decreased to 0.54-fold of Ctrl + NSD (39.06 ± 1.74 µm *vs* 72.42 ± 6.93 µm, *P* = 0.001), 0.61-fold of cKO + NSD (39.06 ± 1.74 µm *vs* 64.32 ± 1.69 µm, *P* = 0.023), and 0.57-fold of Ctrl + HSD (39.06 ± 1.74 µm *vs* 69.04 ± 5.14 µm, *P* = 0.004) (Fig. 7F). Similarly, the total process length *per* cell was reduced to 0.59-fold of Ctrl + NSD (148.78 ± 16.25 µm *vs* 252.93 ± 19.91 µm, *P* = 0.001), 0.62-fold of cKO + NSD (148.78 ± 16.25 µm *vs* 239.24 ± 18.78 µm, *P* = 0.008), and 0.60-fold of Ctrl + HSD (148.78 ± 16.25 µm *vs* 246.44 ± 9.41 µm, *P* = 0.002) (Fig. 7G).​ Moreover, sholl analysis further revealed a significant decrease in the number of intersections in microglia from both mutant and cKO mice fed with an HSD (*P* = 0.005, cKO + HSD *vs* Ctrl + NSD; *P* = 0.009, cKO + HSD *vs* Ctrl + HSD; Fig. 7H, Fig. S2). Taken together, these findings indicate that neuronal Sik1 suppresses microglia activation in the PVN in response to a high-salt diet.

## Discussion

**Fig. 8:**
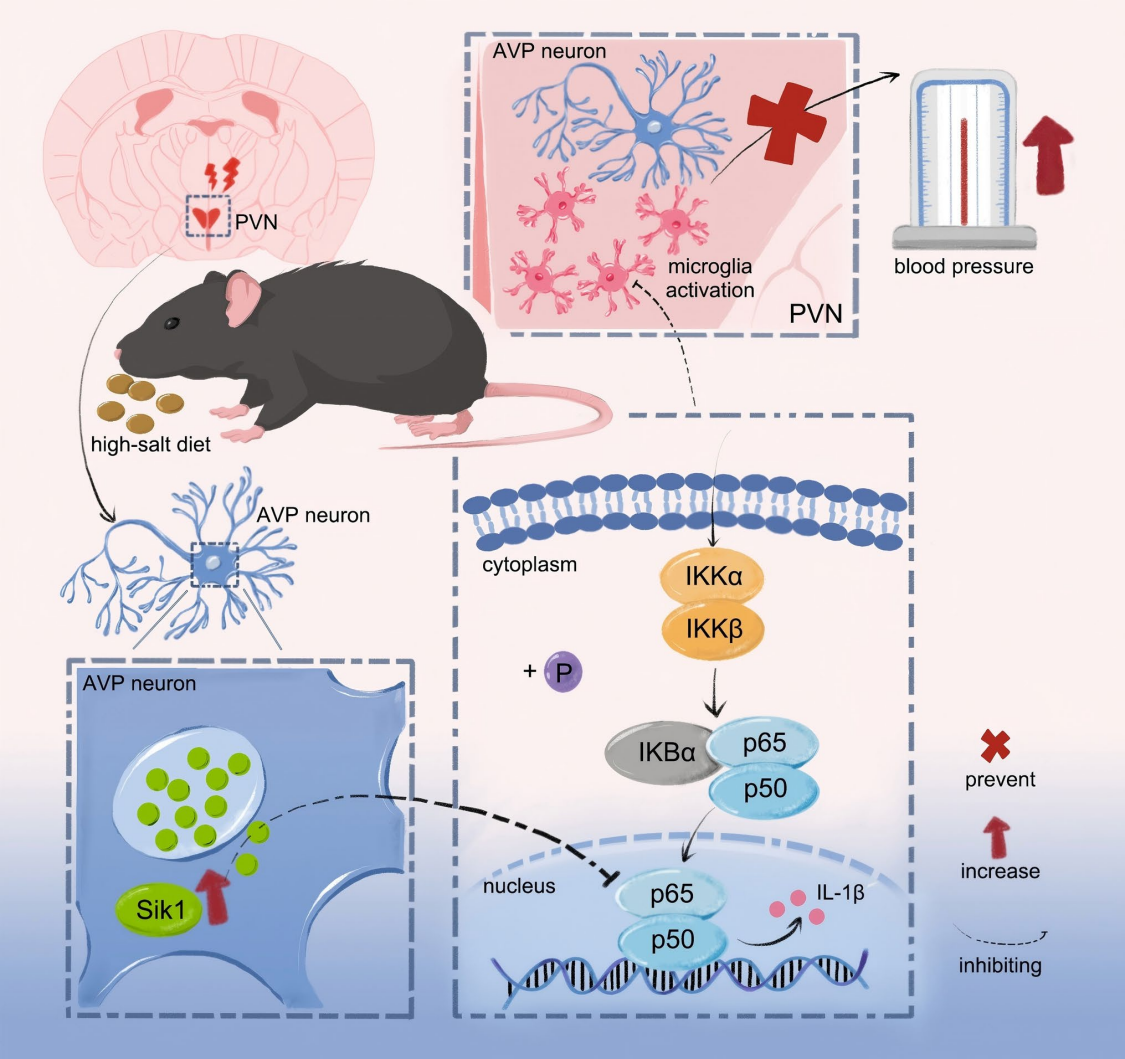
Schematic diagram for the role and its molecular mechanisms of Sik1 in PVN neurons in preventing high salt-induced BP elevation

Excessive salt intake is considered a risk factor for elevated blood pressure [12, 13], though the precise neurogenic mechanisms underlying the pathophysiological process remain obscure. In this work, we provide evidence that Sik1 mediates the PVN’s regulation of BP under HSD conditions. Firstly, we observed an increase in Sik1 expression in the PVN following HSD. Secondly, the results showed increased BP in *Sik1*^–/–^ mice fed an HSD. Thirdly, *Nestin-Cre;Sik1*^–/–^ mice also exhibited elevated BP under high salt intake. As well as, AAV-Cre-mediated selective ablation of Sik1 in the PVN neurons was sufficient to cause salt-induced BP elevation. Notably, our integrated single-nucleus RNA sequencing and KEGG pathway analysis localized Sik1 expression predominantly to AVP neurons in the PVN. Further analysis revealed a significant association between Sik1 expression in the AVP neuronal subpopulation and the NF-κB signaling pathway under HSD conditions. ​Moreover, we found that Sik1 expression leads to inhibition of neuron-centered NF-κB p65 activation in PVN neurons, as well as microglial activation, in response to an HSD. These findings indicate that Sik1 in AVP-positive neurons of the PVN responds to high salt intake and prevent blood pressure elevation, an effect associated with inhibition of the NF-κB p65 signaling pathway and suppression of neuroinflammation (Fig. 8). Our study provides direct evidence that Sik1 in AVP-positive neurons of the PVN contributes to blood pressure elevation under high-salt conditions, offering novel insights into the neurogenic mechanisms underlying blood pressure regulation.

High salt intaking elevates [Na⁺] concentration in both the plasma and CSF [14–16]. The increased CSF [Na⁺] enhances sympathetic nerve activity consequently raising blood pressure [41–43]. This process is initiated when central sodium sensors, notably the Nax channel within the organum vasculosum of the lamina terminalis (OVLT), detect elevated sodium levels and subsequently activate sympathetic outflow via the PVN to rostral ventrolateral medulla (RVLM) pathway [14–16, 44, 45]. Concurrently, at the cellular level, elevated intracellular [Na⁺] activates a critical signaling cascade. Elevated intracellular [Na⁺] promotes Ca^2^⁺ influx through the reverse-mode Na⁺/Ca^2^⁺ exchanger. This Ca^2^⁺ influx activates the phosphatase calcineurin, leading to the dephosphorylation and subsequent nuclear translocation of the transcriptional coactivator TORC1 [46, 47]. Within the nucleus, TORC1 complexes with the cAMP response element-binding protein (CREB), driving the transcription of target genes, including *Sik1* [46]. This mechanistic cascade might provide an explanation for the observed upregulation of Sik1 within PVN neurons under both HSD feeding and acute hypertonic stimulation (Fig. 1B–L). Importantly, selective ablation of Sik1 specifically within the brain, and more precisely targeted to the PVN, resulted in a significant increase in BP under high-salt condition (Figs. 3 and 4). As a component of a cellular sodium-sensing network, studies have reported that Sik1 enhances the catalytic activity of Na⁺/K⁺-ATPase in response to modest elevations in intracellular sodium, thereby preventing abnormal sodium accumulation and contributing to ionic homeostasis [47, 48]. However, it remains unclear whether Sik1 deficiency induces pathological hyperactivity in PVN neurons following a high-salt diet. Future studies should examine how Sik1 in PVN neurons regulates Na⁺/K⁺-ATPase (NKA) activity and neuronal excitability under high-salt conditions. Together, these findings suggest that Sik1 in PVN neurons plays an important role in modulating salt-induced blood pressure elevation.

PVN is a functionally highly heterogeneous nucleus, including diverse neuronal populations that govern integrated physiological responses. Among these, AVP neurons primarily regulate fluid balance and osmotic homeostasis [49, 50], CRH neurons mediate the stress response[24, 51, 52], and OXT neurons contribute to regulation of socio-emotional and socio-sexual behaviors, as well as homeostatic, metabolic, and autonomic responses[53–55]. Previous work has shown that Sik1 in the PVN is involved in hypothalamic–pituitary–adrenal (HPA) axis regulation under chronic stress, where its downregulation promotes CRH transcription via CRTC1 activation [24]. However, the role of Sik1 in other neuronal populations within the PVN remains largely unclear. Interestingly, we observed Sik1 predominantly within AVP-expressing neurons, evidenced by single-nucleus RNA sequencing (Fig. 5E and F). Indeed, AVP neurons critically mediate sympathetic excitation during high-salt stimulation [56–58]. Salt loading enhances AVP neuronal activity and elevates plasma AVP, while PVN vasopressin receptor blockade reduces salt-induced sympathetic excitation and hypertension [56, 57]. Previous research has reported that AVP may contribute to hypertension through several pathways, including sympathetic excitation mediated by vasopressin receptors (V_1a_ receptors) in the PVN region [59], brain RAS hyperactivity via V_2_ receptors [60], and peripheral vasoconstriction induced by V_1_ receptors [61]. Thus, these findings indicate that AVP neurons largely mediate Sik1’s effect on high salt–induced blood pressure elevation.

Previous study has found that overexpression of Sik1 significantly inhibited NF-κB activity in response to lipopolysaccharide stimulation and affected the expression of proinflammatory cytokines [62]. In our work, we found that the NF-κB signaling pathway was enriched in Sik1-expressing AVP neuronal subpopulation under high-salt diet condition, and Sik1 deficiency in PVN neurons resulted in activation of NF-κB p65 (Figs. 5F − G and 6). Previous reports have shown that NF-κB p65 contributes to the cleavage of vasodilatory receptors such as β2-adrenergic receptors and facilitates the generation of angiotensin II type 1 receptors (AT1-R) in neurons, processes linked to the loss of vasodilatory function in arterial smooth muscle [63]. Furthermore, NF-κB blockade would attenuate angiotensin II-induced hypertension in rat [64]. Our findings suggest​ that neuronal Sik1 may be involved in​ the regulation of hypothalamic neuroinflammation in hypertension via the NF-κB p65 signaling pathway. This pathway promotes the transcription of key inflammatory mediators, including TNF-α, IL-1β, and IL-6 [8, 65, 66]. In addition, other signaling pathways may involve in Sik1-positve within AVP neurons after an HSD (Fig. 5G), and can be further validated. Moreover, another key manifestation of hypothalamic neuroinflammation involves bidirectional communication between neurons and microglia. Microglia constantly surveil the neuronal environment through receptors such as P2Y_12_ and GABA [67, 68]. Microglia help maintain circuit stability by modulating synaptic pruning and releasing factors like PDGFB, which enhances neuronal Kv4.3 potassium channel expression and restrains sympathetic hyperactivity [41]. Microglial activation observed in our Sik1-deficient model suggests a disruption in this homeostatic balance (Fig. 7). As a negative regulator of TGF-β signaling [69–71], Sik1 deletion may disrupt TGF-β-mediated homeostatic signaling, thereby contributing to the observed microglial activation. Notably, Gu et al. revealed that in high-salt models, microglia in the PVN become reactive and remodel astrocytes, impairing glutamate clearance, enhancing extrasynaptic NMDA receptor activation, and increasing AVP neuronal activity—resulting in a vasopressin-dependent hypertensive phenotype [72]. Gu et al.’s findings may provide an alternative mechanistic explanation for elevated BP after Sik1 deletion in high-salt model. While microglial activation was observed in our study, it is unclear the specific communicative signals involved are unknown. Future studies should investigate how Sik1 regulates the dialogue between neurons and microglia, which may reveal novel mechanisms for neuroinflammatory components of cardiovascular disease.

Additionally, the central renin–angiotensin–aldosterone system (RAAS) constitutes a classical pathway driving salt-sensitive hypertension. Notably, while peripheral RAAS is inhibited, hypothalamic RAAS activity is specifically upregulated in salt-sensitive hypertension [14, 73]. A study has shown that high sodium intake leads to sustained increases in hypothalamic renin gene expression, despite reduced renal renin expression [74]. Consistent with this finding, salt-sensitive hypertensive patients exhibit elevated ACE and AT_1_ receptor expression in the hypothalamus and brainstem, particularly following activation of brain sodium channels [75]. Blocking these sodium channels attenuates both hypertension and sympathetic overactivity induced by central hypertonic saline load [74, 76, 77]. Interestingly, a study showed that urinary noradrenaline levels were significantly increased in *Sik1*^−/−^ mice following an HSD [78]. Therefore, future work to explore the role of Sik1within this network will be helpful to our mechanistic understanding.

In summary, Sik1 expression in PVN neurons attenuated high salt-induced blood pressure elevation, likely through modulation of the NF-κB p65 signaling pathway. Our findings provide novel insights into the molecular mechanisms of blood pressure regulation under high salt conditions and advance our understanding of the neurogenic basis of salt‑induced hypertension.

## Supplementary Information

Below is the link to the electronic supplementary material.

Supplementary file1 (PDF 495 KB)

Supplementary file2 (PDF 404 KB)

## Data Availability

The datasets used and/or analyzed during the current study are available from the corresponding author on reasonable request.

## Abbreviations

BP: Blood pressure
PVN: Paraventricular nucleus
Sik1: Salt-inducible kinase 1
HSD: High-salt diet
NSD: Normal-salt diet
SNS: Sympathetic nervous system
AMPK: AMP-activated protein kinase
SBP: Systolic blood pressure
GAPDH: Glyceraldehyde-3-phosphate dehydrogenase
NF-κB: Nuclear factor kappa-B
IĸB-α: Inhibitor of kappa-B-alpha
Iba1: Ionized calcium-binding adapter molecule 1
IL-1β: Interleukin-1β
KEGG: Kyoto encyclopedia of genes and genomes
CSF: Cerebrospinal fluid
AVP: Arginine vasopressin
OXT: Oxytocin
CRH: Corticotropin-releasing hormone
RAAS: Renin-angiotensin-aldosterone system
