# Supplementary material for: Neuronal Sik1 in the Hypothalamic Paraventricular Nucleus Decreases Blood pressure Elevation Following a High-Salt Diet
Source: Mol Neurobiol. 2026 Jan 16;63(1):368. doi: 10.1007/s12035-026-05666-6 (PMC12811292; doi:10.1007/s12035-026-05666-6)
Supplement: Supplementary file 1 — Supplementary file1 (PDF 495 KB) [file 12035_2026_5666_MOESM1_ESM.pdf]

Supplementary Materials

Tan_SI Figure 1

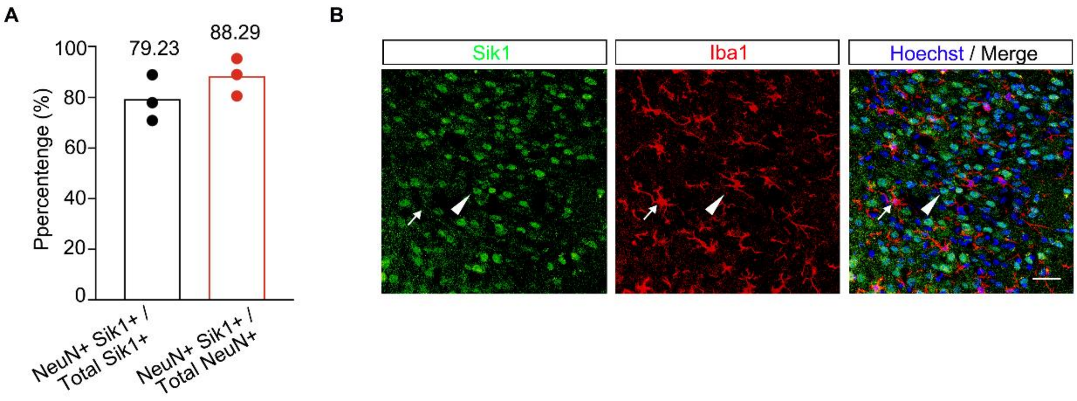

Figure S1. The distribution of Sik1 protein in PVN cell types of mice. (**A**) The co-localization percentage of Sik1 protein and NeuN-positive neurons in the PVN. (**B**) The distribution of Sik1 protein in Iba1-positive cells in the PVN by immunostaining. Iba1 was used as a protein marker for microglia. Hoechst was used for counterstaining of the nucleus (blue). Arrowheads indicate Iba1+ cell; Arrows indicate Iba1- cells. Scale bar, 30μm.

Tan_SI Figure 3

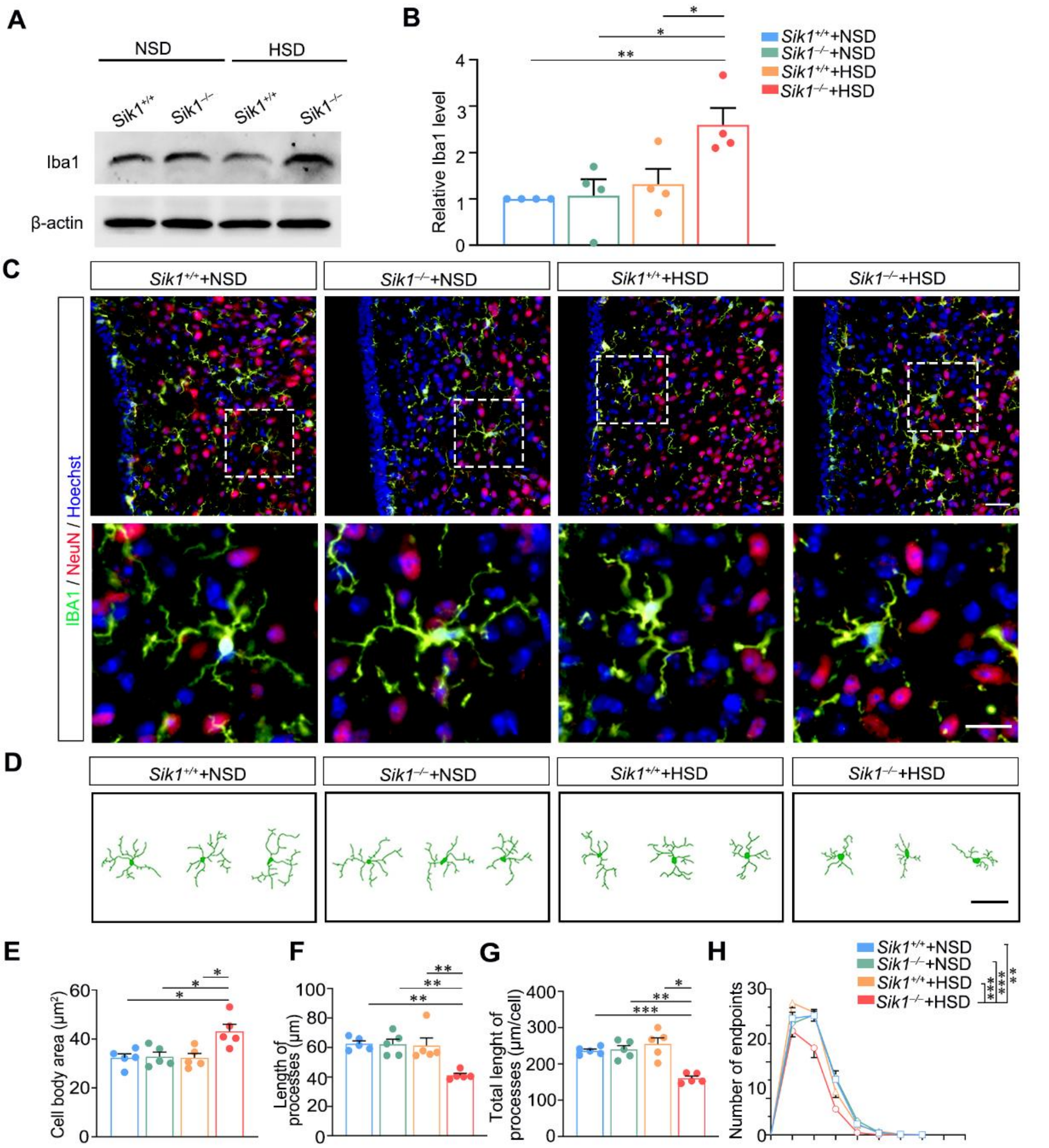

Figure S2. Microglial activation in the PVN of *Sik1*$^{-/-}$ mice after HSD. (**A**) Representative immunoblot bands and quantitative analysis of Iba1 expression in the PVN across the four groups. β-actin was used as a loading control. (**B**) Statistical analysis of Iba1 expression. (**C**) Representative immunofluorescence images of Iba1 in the PVN across the four groups. Boxed frame indicate microglial cells. Scale bar, 30 μm. (**D**) Neurolucida tracings of Iba1-labeled microglial cells. Scale bar: 30 μm. (**E**) Statistical results of body area of microglia within the PVN. (**F**) Statistical results for the average length of microglial processes. (**G**) Statistical results for the total length of processes per microglia. One-way ANOVA with *post hoc* Dunnett's test. (**H**) Sholl analysis was performed to quantify the number of intersections at increasing radial distances from the microglial soma in the PVN. $n = 5$ *per* group. Two-way ANOVA with *post hoc* Bonferroni test. For each mouse, at least 15 of the largest microglial cells

within a 40,000 μm² hypothalamic area were analyzed. $*P < 0.05$, $**P < 0.01$, $***P < 0.001$. Data are expressed as mean ± SEM.
