# Supplementary material for: Neuronal Sik1 in the Hypothalamic Paraventricular Nucleus Decreases Blood pressure Elevation Following a High-Salt Diet
Source: Mol Neurobiol. 2026 Jan 16;63(1):368. doi: 10.1007/s12035-026-05666-6 (PMC12811292; doi:10.1007/s12035-026-05666-6)

**Figure 1**

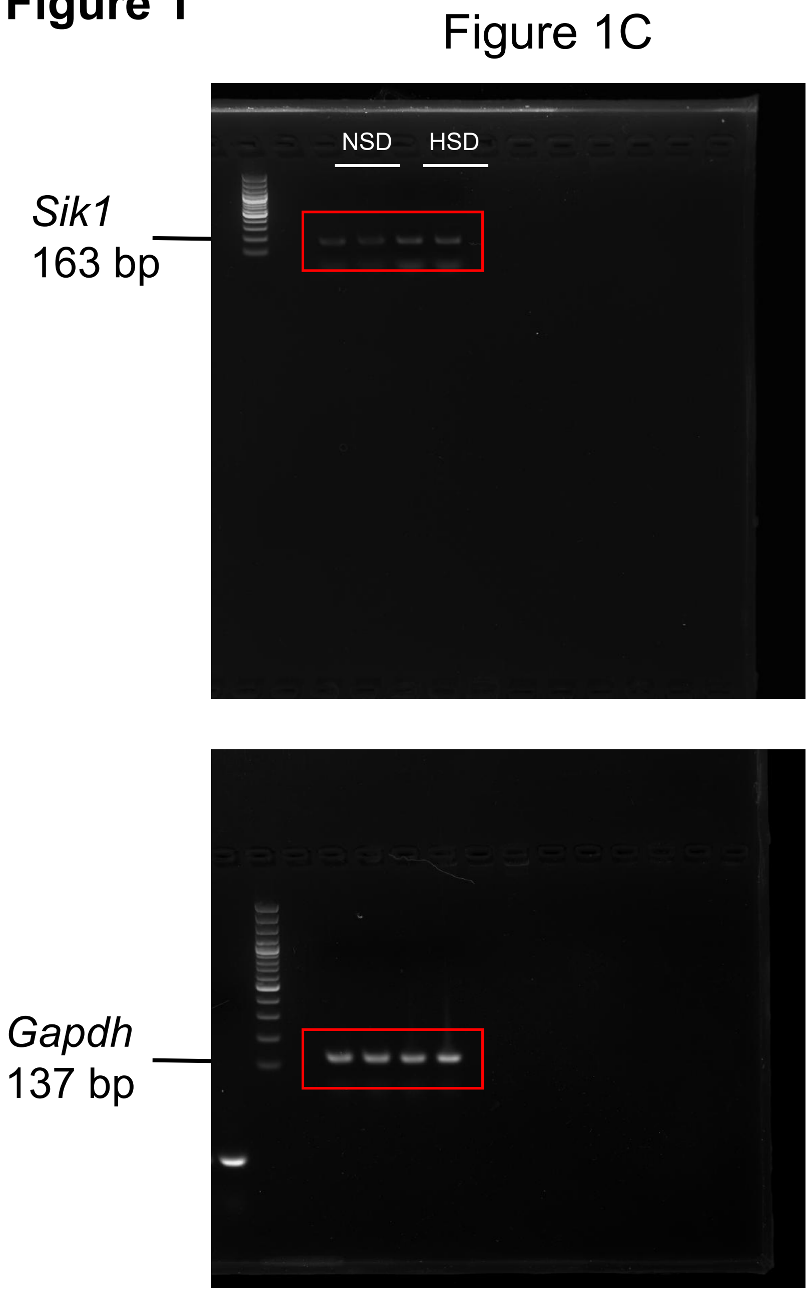
Figure 1C
NSD
HSD
*Sik1*
163 bp
*Gapdh*
137 bp

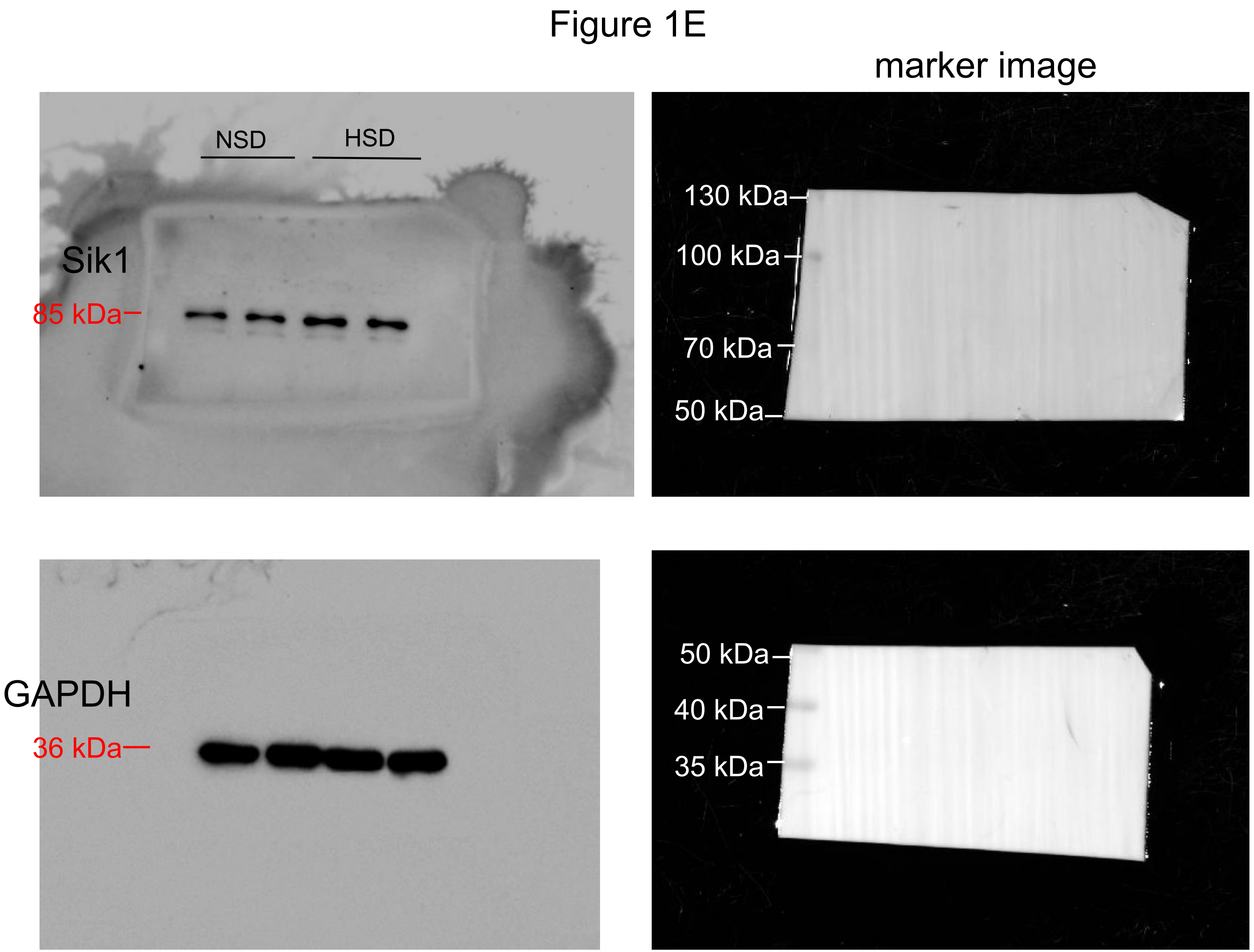
Figure 1E
marker image
NSD
HSD
Sik1
85 kDa
130 kDa
100 kDa
70 kDa
50 kDa
GAPDH
36 kDa
50 kDa
40 kDa
35 kDa

**Figure 2**

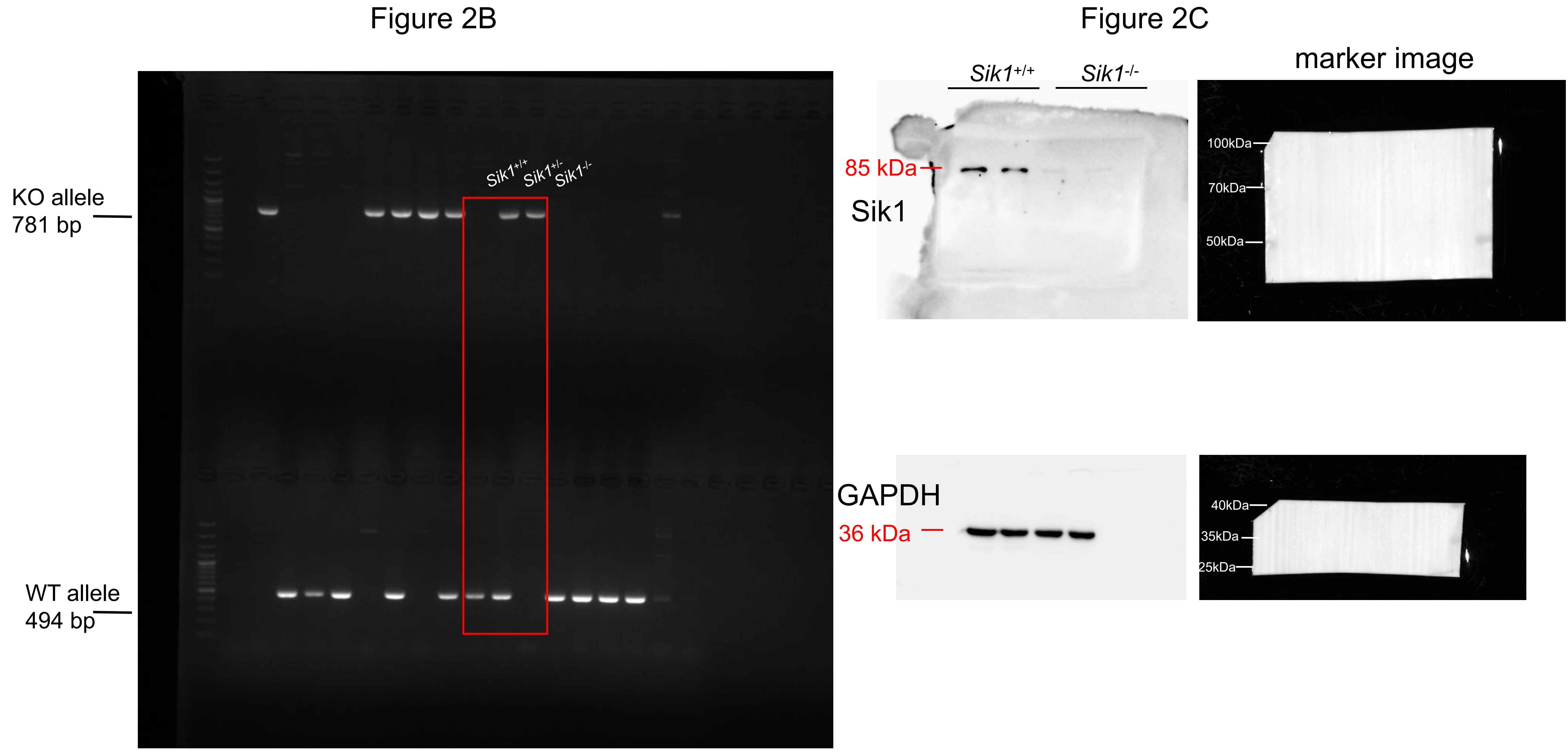
Figure 2B
KO allele
781 bp
WT allele
494 bp
Sik1+/+
Sik1+/-
Sik1-/-
Figure 2C
Sik1+/+
Sik1-/-
marker image
85 kDa
Sik1
100kDa
70kDa
50kDa
GAPDH
36 kDa
40kDa
35kDa
25kDa

**Figure 3**

Figure 3B

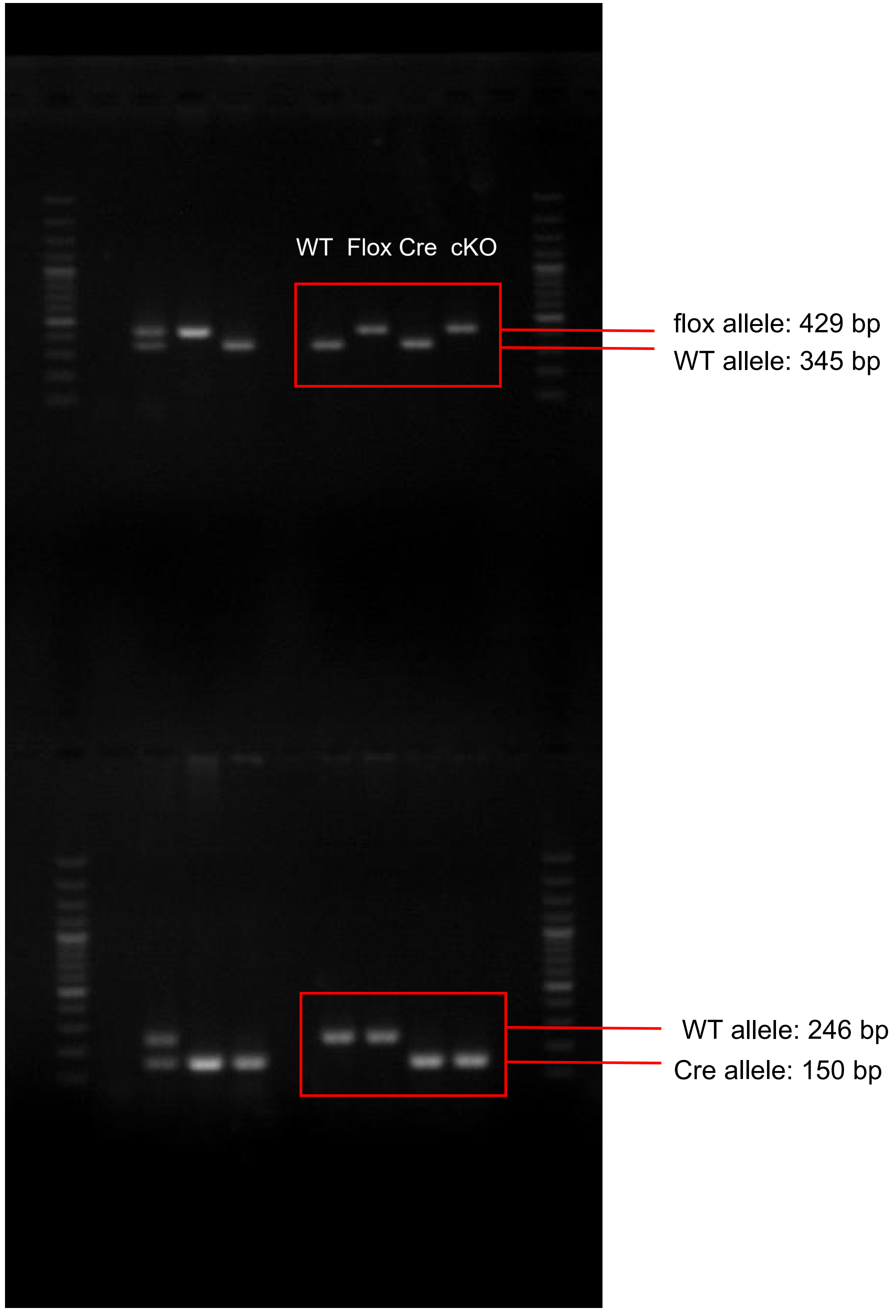


Figure 3C

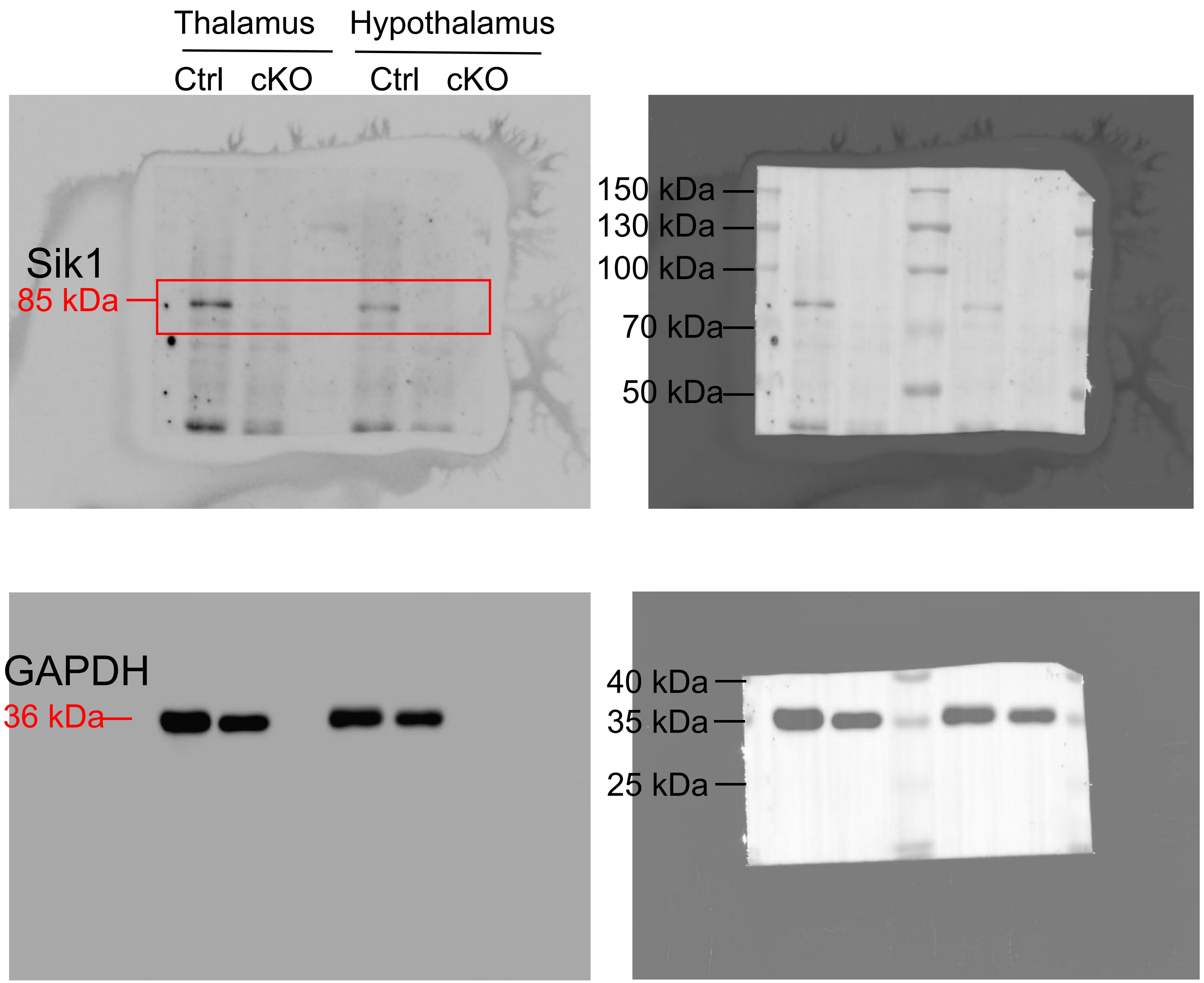

**Figure 3D**

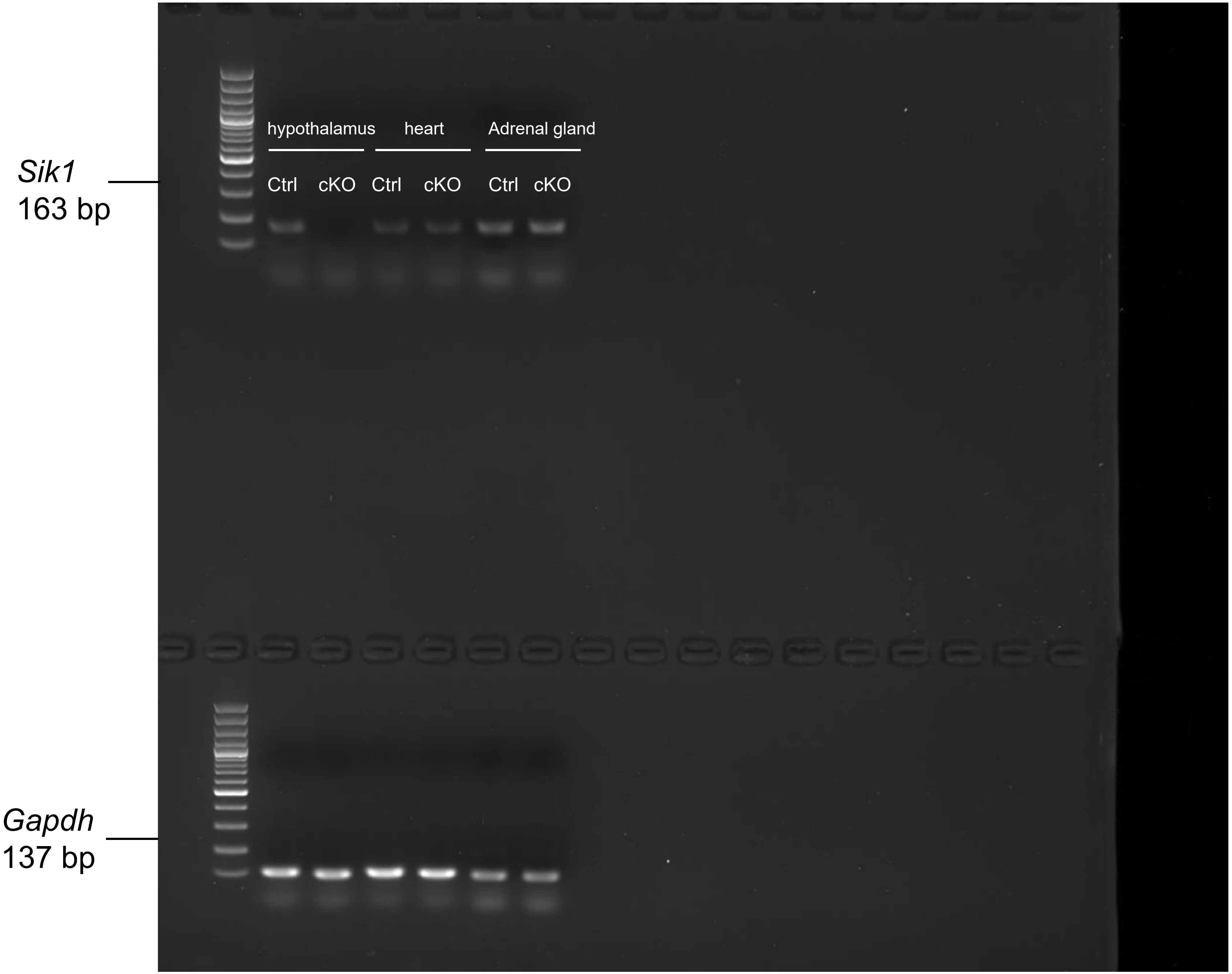


*Sik1*
163 bp

*Gapdh*
137 bp

**Figure 6A**

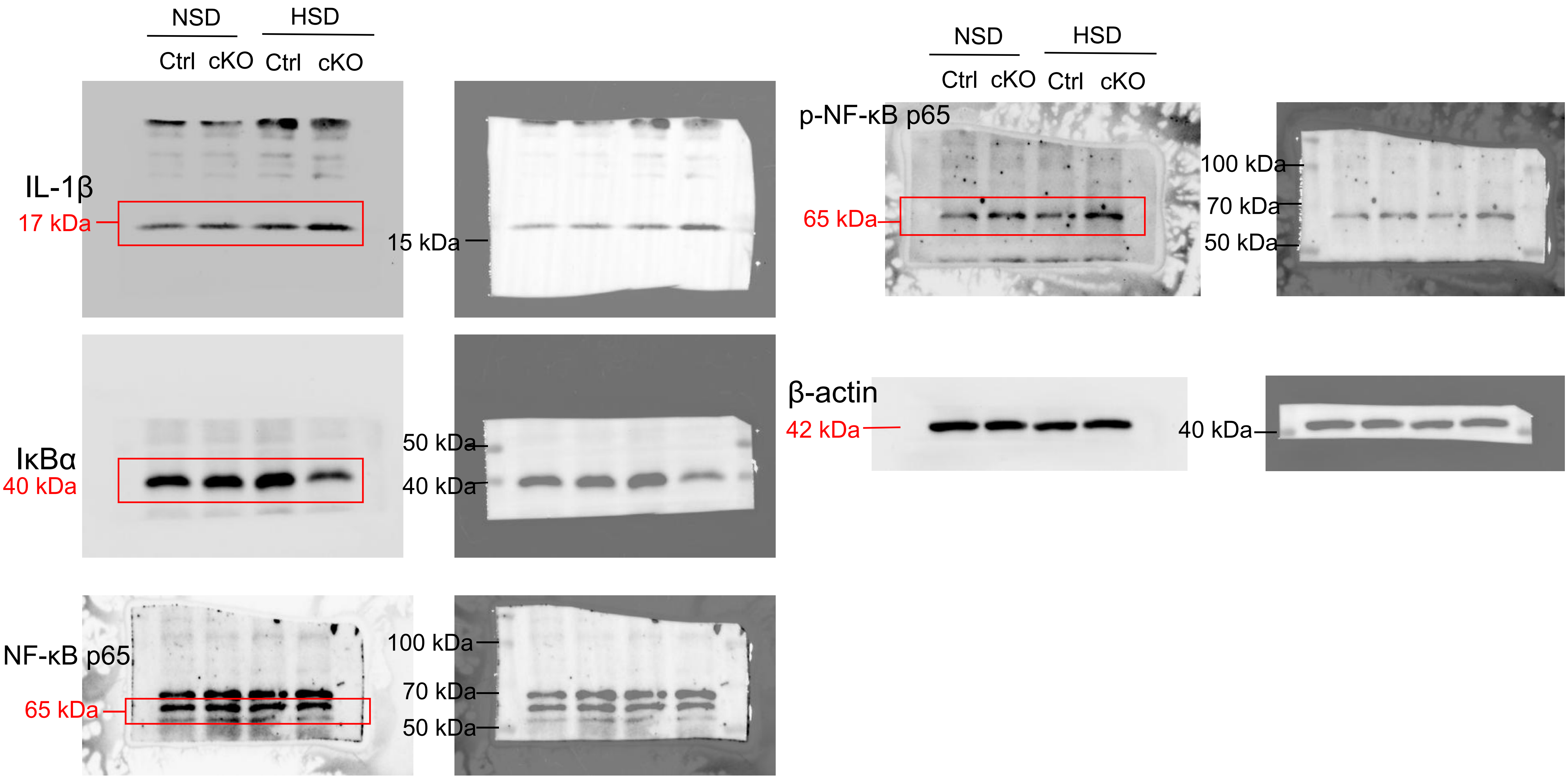
NSD
HSD
Ctrl
cKO
Ctrl
cKO
IL-1β
17 kDa
15 kDa
IκBα
40 kDa
50 kDa
40 kDa
NF-κB p65
65 kDa
100 kDa
70 kDa
50 kDa
p-NF-κB p65
65 kDa
100 kDa
70 kDa
50 kDa
β-actin
42 kDa
40 kDa

**Figure 7**

Figure 7A

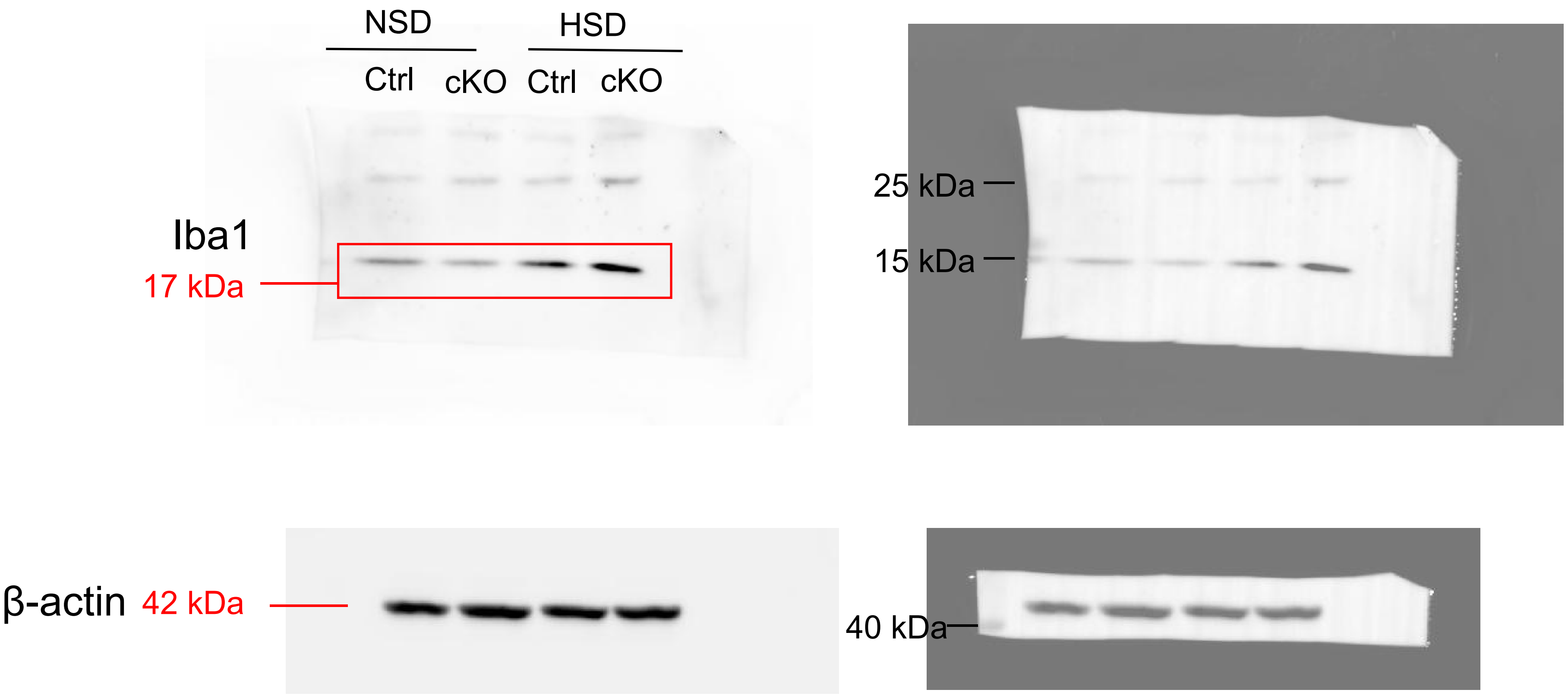

# SI Figure2

## SI Figure 2A

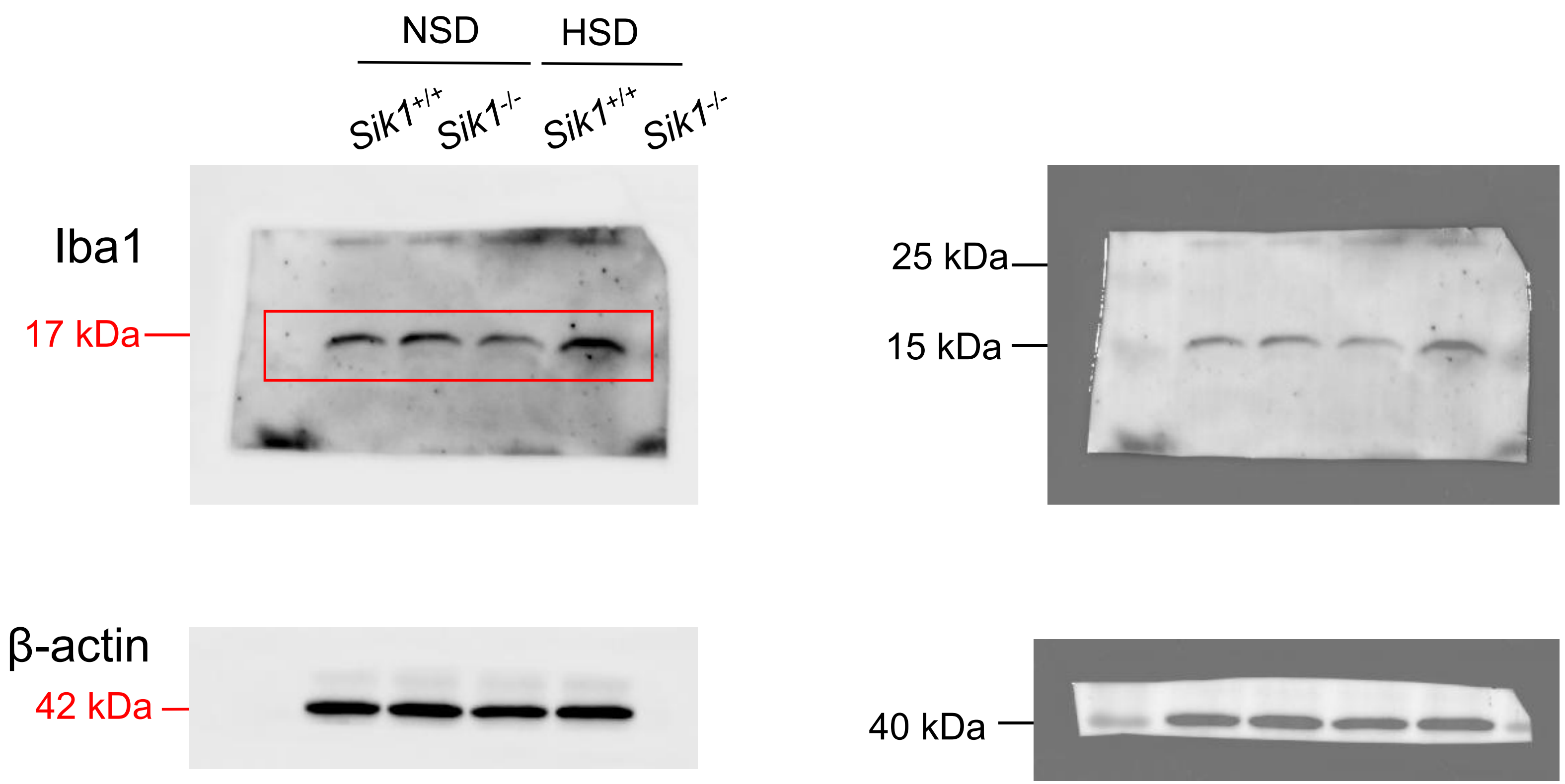

Supplement: Supplementary file 2 — Supplementary file2 (PDF 404 KB) [file 12035_2026_5666_MOESM2_ESM.pdf]
